# Contribution of social factors to maternal deaths in urban India: Use of care pathway and delay models

**DOI:** 10.1371/journal.pone.0203209

**Published:** 2018-10-09

**Authors:** Manmeet Kaur, Madhu Gupta, Vijin Pandara Purayil, Monica Rana, Venkatesan Chakrapani

**Affiliations:** Department of Community Medicine and School of Public Health, Postgraduate Institute of Medical Education and Research (PGIMER), Chandigarh, India; London School of Economics and Political Science, UNITED KINGDOM

## Abstract

This paper uses care pathway and delay models to better understand the possible social reasons for maternal deaths in a city with good public and private health infrastructure. The findings can inform programmes to reduce maternal mortality. During 2007–15, 136 maternal deaths were reported in Chandigarh, India. Using World Health Organisation’s verbal autopsy questionnaire, interviews were conducted with primary caregivers of 68 (50%) of the 136 deceased women, as majority of the families had returned to their native places. We used process-tracing techniques to construct the care pathways and identify delays, and explored open-ended responses using thematic analysis. The mean age of the deceased women was 27 years, 51% resided in slums, 32% were primigravida, 25% had their deliveries assisted by traditional birth attendants, and 23% had Caesarean section. Eight percent died at home, and 54% died in tertiary level facilities. Post-partum haemorrhage (26.5%), and complications of puerperium (25%) and labour/delivery (14.7%) were the reported medical causes. Male child preference and norms for home delivery were identified as the distal socio-cultural causes. Individual and family level factors included: shame on multiple pregnancies; fear of discrimination from providers; past successful deliveries at home leading to overconfidence and not seeking institutional care; and lack of awareness about family planning, antenatal care, and danger signs of pregnancy. Healthcare system factors were: non-availability of senior doctors at the time of consultation in the emergency that delayed initiation of immediate treatment, and lack of availability of life-saving equipment due to patient load. Empirical evidence was found on social causes of maternal deaths, which could have been prevented by appropriate actions at individual, family, societal, institutional and policy levels. This study identified potential preventable causes of primarily social origin, which could help in taking actionable steps at several levels to further reduce maternal deaths in India.

## Introduction

Pregnancy is a vulnerability that put a woman at risk of dying [1]. After the commitment to achieve the millennium development goals (MDGs), since 1990, a sharp maternal mortality decline of 43% was reported globally [2]. During the same period, many countries in Sub-saharan Africa recorded 50% reduction in maternal mortality. By the end of 2015, by which the MDGs were supposed to have been achieved, the MMR in developing counties was 239 per 100,000 live births versus 12 per 100,000 live births in developed countries highlighting the obvious maternal health inequalities globally. Developing countries share 99% of the global maternal deaths.

The decline in maternal mortality ratio (MMR) among Asian and North African countries was only 2.3% per year between 1990 and 2015 [2]. Efforts to achieve MDGs were followed systematically in some countries, and there was an annual decline of above 5.5% in maternal mortality between the years 2000 and 2010, which was essential to achieve the MDG-5. However, this decline was not uniform across countries. This can be explained by the existing maternal and child health policies and programs that have been extensively reviewed in several low and middle-income countries (LMIC) by the Partnership for Maternal Newborn and Child Health. It has led to a better understanding on how some countries could accelerate progress in reduction of preventable maternal and child deaths [3].

There are 10 fast-track countries including Bangladesh, Nepal, Cambodia, China, Egypt, Ethiopia, Lao PDR, Peru, Rwanda and Vietnam [4], that have invested in high-impact health interventions, such as quality care at birth, immunization and family planning. It also identified the role played by diverse sectors such as education, sanitation and water supply, and employment, in creating and sustaining an environment that supports the work of health systems and health partners while in other LMICs a comprehensive approach is still lacking. Moreover, there is a need to identify specific social and cultural factors that might be leading to maternal deaths.

Sustainable Development Goal 3 (SDG-3) has a target to reduce the global MMR to less than 70 per 100,000 births by 2030 [5]. The maternal health inequalities are not only between countries, but within countries, and between rich and poor women residing in villages, cities and slums [1, 6–8]. Layers of maternal health inequalities can be traced in the provision of maternal care services as well as across socioeconomic gradient in India [9, 10]. These inequalities lead to conditions that make some women more vulnerable to death than others. In developing countries, including India, early age at marriage and preference to have a male child (in turn resulting in multiple pregnancies) are some of the social conditions that increase lifetime risk of death due to pregnancy among poor women. This lifetime risk of maternal deaths is 1 in 4900 pregnant women in developed, 1 in 180 in developing and 1 in 54 in underdeveloped countries [2].

Biomedical causes and health system conditions that lead to maternal deaths are well recognised [11]. Such studies have contributed in reducing maternal deaths that occur due to complications during and following pregnancy and childbirth (such as post-partum haemorrhage, sepsis, obstructed labour). The health-care systems were geared to prevent or manage complications and health system now successfully prevent most of such deaths [12, 13]. However, it is not sufficient to prevent all the maternal deaths, as there are other factors which play an important role in determining who will survive and who will not [14].

The ‘three delays’ model (delay within home, on the way to a health facility and within a health facility) in receiving maternal care that are available in literature do not provide a holistic view of social causes that lead to death of pregnant women in developing countries [15]. Lots of efforts that have been made to reduce delays by implementing maternal death review in the Southeast Asian Region have reduced maternal mortalities, but the pace of such decline is slow. Hence, the underlying causes that are responsible for delays still need to be understood and addressed [16]. Moreover, the delay models have been tested through empirical, primarily quantitative studies [17, 18]. So far, the policy recommendations have been that all pregnant women need access to antenatal care in pregnancy, skilled care during childbirth, and care and support in the weeks after childbirth. The same guidelines are being followed in India, even then pregnant women continue to die.

The socio-economic and cultural conditions that often contribute to delays need to be understood and explained to prevent these untimely maternal deaths, especially, in urban areas despite the availability of best health facilities within reach. The purpose of this study was to explore the underlying causes leading to maternal deaths between the years 2007 and 2015.

## Methods

### Study settings

This study was conducted in Chandigarh, a city in North India, which has a population of 1,055,450 (60% urban, 37% slums and 3% rural), as per the 2011 Indian census [19]. We chose to conduct this study in Chandigarh as it presents a unique situation. On one hand, it has one of the best health infrastructures in the country with multiple health care facilities in public (44 primary, 5 secondary care and 2 tertiary care health facilities) and private sector that are spread over a small geographical area (114 sq/km), good all weather roads and connectivity, highest per capita income (USD 1000), and first rank in human development index in the country [20]; and on the other hand 30% of its population are migrants, who are vulnerable and reside in slums/rehabilitative colonies [21]. Another characteristic of this city that makes it an ideal candidate for this study as maternal mortality rate in this city has remained at 100–120 per 100,000 live births from 2007 to 2015 [22], in spite of the increase in institutional delivery rate from 73.6% (DLHS, 2007–08) [23] to 91.6% (NFHS, 2015–16) [24] during that period.

### Ethics approval

Written informed consent was obtained from all participants. The Institutional Ethics Committee of Postgraduate Institute of Medical Education and Research (PGIMER) reviewed and approved the study.

### Participants and data collection methods

In 2007, the School of Public Health in Postgraduate Institute of Medical Education and Research, Chandigarh, established a maternal death audit system to improve monitorable indicators of reproductive and child health. A total of 136 maternal deaths were reported between 2007 and 2015 as part of this audit, which was independent of the routine maternal death review in the health system. (S1 File). The maternal death audit system included reporting of maternal deaths, along with their residential addresses, from all the delivery points in public health system including 2 tertiary and 4 secondary care hospitals. For the deaths occurring in private health facilities or at home, data were obtained from Family Welfare Bureau through the respective auxiliary nurse midwives of the catchment area, and also from the cremation ground or cemetery. To ensure that no deaths were missed, data were triangulated with maternal deaths recorded in the office of registrar births and deaths in Chandigarh, on a monthly basis. Within 3 to 4 weeks of reported maternal deaths, two graduate-level trained field investigators visited those houses to conduct the verbal autopsy, during 2007–15. Minimum three visits were made to meet the index women’s caregivers. However, in some cases, where the neighbours had told that family members would come back from their native village, 5 to 8 visits were also made. They interviewed the relatives of the deceased who were present at the time of development of illness, accompanied her to the health facilities, and stayed with her till the time of death, to ascertain biological and non-biological causes of maternal deaths. A majority of the caregivers interviewed were husband or parents-in-law. They were taking care of the women when she was pregnant or when she was in labour, and were women taking care of her during the illness, accompanied her to the health facilities, and stayed with her till the time of death. WHO’s standard verbal autopsy questionnaire for maternal deaths was used after obtaining the written informed consent [25]. Out of 136 visits, 68 (50%) verbal autopsies were completed. Reasons for non-completion of verbal autopsies included shifting of families to their native places (32%), wrong address (12%) and locked houses (5%).

Medical causes were ascertained by coding the verbal autopsy questionnaire by two trained medical officers as per international classification of diseases (ICD-10) [26]. In case of discrepancy, opinion of a third trained medical officer was obtained. Social causes including factors or circumstances that led to death were ascertained from the recorded qualitative narrative of the events (n = 68), as perceived by the caregivers in the family. (S2 File). In this paper, we analysed the responses to open-ended questions. That focused on understanding the caregivers’ perceived causes of maternal deaths, i.e., to whom or what they attribute the deaths to; and also to the various circumstances in the background at individual, family and societal levels that could have contributed to maternal deaths.

### Analyses

Responses to open-ended questions and field notes from interactions with caregivers were translated into English by professional translators. We randomly selected 20% of the translated text to check for accuracy in translation by comparing them with the original verbal autopsy questionnaires. Data were analysed using techniques derived from narrative thematic analysis [27] and process tracing method [28] to trace potential causal pathways that led to maternal deaths. We used a combination of ‘pre-determined’ or ‘a priori’ codes as well as codes that emerged during the analysis (emergent codes) using NVivo-10. The qualitative findings are presented using a combination of delay model [15] (delay within home, on the way to a health facility and within a health facility) and care pathway model [29], the two dominant ways by which causes of maternal deaths have been described in the literature.

Descriptive analysis of socio-demographic variables, and delays in maternal care were conducted by using IBM SPSS-21. Differences in key demographic variables (age and area) between deceased women whose caregivers were contacted for verbal autopsies (n = 68) and those who were not contacted were examined using Chi-square test (for categorical variables) or a t-test (for continuous variables) to check whether there were any significant differences between those two groups.

We had estimated the average time and or distance of the residences of the deceased pregnant women to the nearest first level health care facilities.

## Results

A total of 136 maternal deaths were reported during 2007–15 in Chandigarh. About half of the deaths were reported from urban slum areas (47.1%); more than a quarter (27.9%) from rural areas and another quarter (25%) from urban areas of Chandigarh. The mean age of the deceased women, in the 68 verbal autopsies, was 27 years (SD 5). About half of the deaths were reported from the urban slum areas (51%); another quarter (26.5%) from urban city; and less than a quarter (22.1%) from the rural areas of Chandigarh. One-thirds (36.8%) of these women were illiterate. About half of them were living in the rented (20.5%) and temporary houses (25%). Except two women (3%) who were widowed, rests were living with their husband at the time of delivery/pregnancy. About 32% pregnant women were primigravida, 29.4% delivered at home, 25% were delivered by traditional birth attendants, 60.3% delivered vaginally and 23.5% had caesarean section.

About two third (67.6%) women died after delivery in the postnatal period. Eight percent died at home, while 54% in the tertiary care hospital. (Table 1). There was no statistical significant difference in demographic characteristics like age (p = .72) and area of living (p = .52), between the deceased women whose caregivers were contacted (n = 68) for this study and those who were not (n = 68).

**Table 1:** Sociodemographic profile and other characteristics of women who died of pregnancy-related causes in Chandigarh, between 2007 and 2015 (n = 68/136).

Variables		
Age in years: Mean (SD)	27.1 (4.9)	
	*n*	*%*
**Education**		
Illiterate	25	36.8
Primary	15	22.1
Secondary	18	26.5
Higher Secondary	8	11.8
Don't Know/missing	2	3
**Marital Status**		
Married/Living with a partner	66	97.1
Widowed	2	2.9
**Residential Status**		
Permanent	33	48.5
Rented/Temporary	35	51.5
**Area of living**		
Urban	18	26.5
Slum	35	51.5
Rural	15	22.1
**Occupation**		
Homemaker	62	91.1
Labourer	4	5.9
Salaried employee	2	3
**Pregnancy (Range: 1 to 8)**		
Experienced (Second or more)	47	69.1
First-time pregnancy	21	30.9
**Place of delivery**		
Institutional	35	51.4
Home	22	32.4
Undelivered	11	16.2
**Type of delivery**		
Vaginal Delivery	41	60.3
Caesarean Section	16	23.5
Undelivered	11	16.2
**Delivery assisted by**		
Doctor	28	41.2
Nurse/Midwife	7	10.3
Traditional Birth Attendant	17	25
Self	3	4.4
Undelivered	11	16.2
Others*	2	3
**Time of maternal death**		
Before delivery (antenatal)	11	16.2
During delivery (perinatal)	5	7.4
After delivery (postnatal)	52	76.5
**Previous history of home delivery**	8	11.8
**Place of death**		
Home	6	8.8
Primary care hospital	1	1.5
Secondary care hospital	7	10.3
Tertiary care hospital	47	69.1
Private hospital	1	1.5
On the way to health facility or death happened before reaching the higher facilities	6	8.8

*Neighbours

Sixty-three (92.6%) pregnant women died due to direct and 6 (7.35%) due to indirect medical causes (Table 2). Based on the ICD-10 criteria, the most common direct biological cause of maternal death was post-partum haemorrhage (26.5%); followed by complications of puerperium (25%) like puerperal sepsis (8.8%), obstetric embolism (10.3%), eclampsia (6%); and, complications of labour and delivery (14.7%). Indirect medical causes (n = 57) included tuberculosis, fever, and influenza etc.

**Table 2:** Medical causes of maternal deaths as per ICD-10 coding.

S. No.	Medical causes of maternal death	ICD-10 CODE	n = 68	%
A.	**Direct Causes**		**63**	**92.6**
1.	**Post-Partum Hemorrhage**	**O72**	**18**	**26.5**
2.	**Complications predominantly related to puerperium**		**17**	**25.0**
	Obstetric embolism	O88	7	10.3
	Puerperal sepsis	O85	6	8.8
	Puerperal Sepsis and Obstetric embolism	O85 & O88	1	1.5
	Venous complications and hemorrhoids in the puerperium	O87	2	2.9
	Complications of the puerperium, not elsewhere classified	O90	1	1.5
3.	**Complications of labour and delivery**		**10**	**14.7**
	Other obstructed labor	O66	2	2.9
	Labor and delivery complicated by intrapartum hemorrhage, not elsewhere classified	O67	1	1.5
	Other obstetric trauma	O71	1	1.5
	Retained placenta and membranes, without hemorrhage	O73	1	1.5
	Other complication of labor and delivery, not elsewhere classified	O75	5	7.35
4.	**Oedema, proteinuria and hypertensive disorders**		**6**	**8.8**
	Pre-existing hypertensive heart disease complicating pregnancy, childbirth and the puerperium	O10.1	1	1.5
	Pre-eclampsia	O14	1	1.5
	Unspecified pre-eclampsia	O14.9	1	1.5
	Eclampsia and Puerperal sepsis	O15 & O85	1	1.5
	Unspecified maternal hypertension	O16	1	1.5
	Gestational edema and proteinuria without hypertension	O12	1	1.5
5.	**Pregnancy with abortive outcome**		**2**	**2.9**
	Failed attempted termination of pregnancy	O07	1	1.5
	Embolism following failed attempted termination of pregnancy	O07.2	1	1.5
6.	**Maternal care related to fetus and amniotic cavity**		**4**	**5.9**
	Multiple gestation, unspecified	O30.9	2	2.9
	Antepartum hemorrhage, not elsewhere classified	O46	1	1.5
7.	**Other obstetric complications**		**6**	**8.8**
	Maternal infectious and parasitic diseases classifiable elsewhere but complicating pregnancy, childbirth and the puerperium	O98	4	5.9
	Other maternal diseases classifiable elsewhere but complicating pregnancy, childbirth and the puerperium	O99	1	1.5
	Diseases of the circulatory system complicating pregnancy, childbirth and the puerperium	O99.4	1	1.5
B.	**Indirect Causes**		**5**	**7.35**
8.	Milliary tuberculosis, unspecified	A19.9	2	2.9
9.	Influenza due to certain identified influenza viruses	J09	1	1.5
10.	Fever of other and unknown origin	R50	1	1.5
11.	Person encounters health services in circumstances related to reproduction	Z30	1	1.5

**Fig 1:**
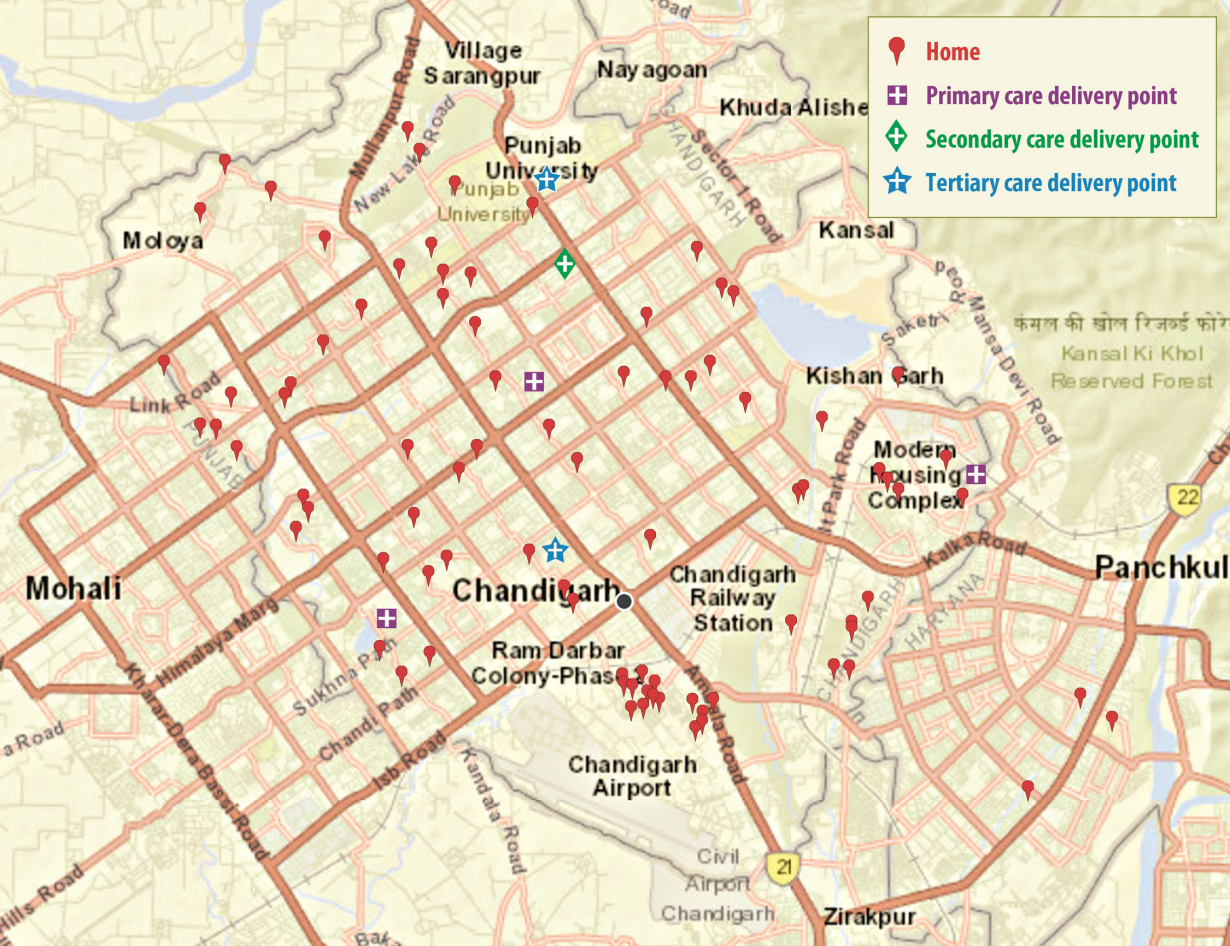
Geographical distribution of maternal deaths in UT Chandigarh.

Fig 1, shows the map of Chandigarh [30] with primary, secondary and tertiary health care delivery points, and the areas in which deceased pregnant women resided. The urban health care facilities in Chandigarh include tertiary care hospitals (2), district or secondary level hospital (1), civil hospitals (primary level delivery points) (3), urban primary health centres (3) and dispensaries (26). In rural area, there are 8 dispensaries, 7 alternative medical centres and 17 sub-centres. In the study sample, the average distance from home to first accessed health care facility was 7.6 km. Most of the maternal deaths were reported within 10 km radius of the city. However, most of these deaths were clustered in slum areas (51.5%), where most migrants and people from lower income group resided.

Themes, including a combination of factors at the level of care (individual, family and type of health care facilities) and where delays occurred (at home, transport or health care facilities), are summarised below:

### Individual and family level factors

#### Delay in deciding to seek care from a proper health care provider/facility

The delay in seeking care was measured on the basis of the time taken from the moment when either the woman or her family realized that there was a problem/complication to reach an appropriate health care facility. Out of the 54 women who delivered vaginally (41/57; 71.9%), more than one third (22/57; 38.6%) delivered at home. The moment complications arose (for example, abdominal pain or bleeding), among those who delivered at home, they sought the help of traditional birth attendants or a private doctor. In 10 of 68 cases decision to take the women to a health facility was delayed by at least 1 hour to 2 days, as the family members tried to get medicines from a local doctor or pharmacy, before taking her to a medical facility. Apparently, this delay might have decreased the chances of survival.

*“Baby boy was born and everything was fine*. *On the second day she complained about having pain in legs*. *We got some medicine from a doctor in a dispensary*, *but she didn't get relief so we took her to a referral hospital [secondary care district hospital]*. *They said that patient was brought dead*. *She died on the way*.” (Notes by the interviewer, Maternal Death ID: 1; died of Obstetric embolism; Age-21; primigravida; rural resident; year 2007)

#### Inadequate ANC and underestimation of danger signs

Some women apparently did not regularly go for care and/or they were not aware of or ignored certain danger signs (like pain in legs and swelling in neck), which might have alerted them to seek a proper medical care at the earliest. For example, 10% did not register for antenatal care in public or private hospitals. Discussions with relatives indicated that this decision might have been influenced by past successful delivery experiences at home, as most of those women who did not register for ANC or did not complete the required ANC visits had a history of previous delivery at home.

The number of all-time pregnancies ranged between 1 and 8, with 42 women (61.7%) having had at least two or more pregnancies. A few relatives opined that shame on multiple pregnancies could have been a reason for not registering for ANC, as those women might have anticipated discrimination or disrespectful behaviour from health care providers. Similarly, it seems that previous negative experiences with health care facilities could have also prevented women from registering for ANC.

*“Before this child*, *she had 5 children that were born normal at home in UP [Uttar Pradesh]*. *In this pregnancy*, *she did not register for pregnancy*, *neither she had taken any tablets (iron folic acid tablets) nor any injection (tetanus toxoid)*. *In nearby private hospital she had consulted a private nurse and her husband was not sure about whether the nurse had given her any injection or not*.” (Notes by the interviewer, Maternal Death ID: 37; died of postpartum haemorrhage; Age-35; multigravida (6); urban slum resident; 2012)

In another case narrated about the underestimation of danger signs:

*“Delivery was conducted at home and relatives were present at the time of delivery*. *Dai was called after delivery*. *Delivery was conducted at 3*.*30 am*. *At the time of delivery patient didn't have any problem and the delivery was normal*. *Severe bleeding was there after delivery and she became pale*. *Dai was called and Dai told that she was okay*. *One hour after delivery there was severe abdominal pain and dai suggested us to take her to the doctor*.*”* (Notes by the interviewer, Maternal Death ID: 13; died of postpartum haemorrhage; Age-33; multigravida (5); urban slum resident; year 2009)

#### Lack of family or partner support

A few incidents were reported in which in-spite of the proper antenatal care, due to non-availability of family support at the time of onset of labour, proper medical care during child birth could not be sought. In one such case, women had to rely on the neighbourer at the time of onset of labour at home, who had brought a traditional birth attendant (*dai)* for delivery. She apparently did not assess the condition of the mother properly and conduct the delivery in aseptic conditions. Thus in this case, due to *dai’*s intervention and possible injury while delivery might have led to the death.

*This was her first pregnancy*. *She used to go to secondary care hospital for check-ups*. *Card was prepared*. *She had no problem*. *During starting of 7th month pain started and was severe*. *Nobody was at home so were not able to go to hospital*. *So a local dai was called*, *and she had checked her*, *and told that delivery can be done at home and pressed her*. *Baby was delivered*. *Baby was still birth*. *Dai had inserted her hand to clear the placenta without wearing gloves*. *She was screaming and suddenly half of placenta came out and half remained attached to her*. *She was saying again and again about pain*, *and died*. (Notes by the interviewer, Maternal Death ID: 19; died of other obstetric trauma; Age-24; primigravida; urban slum resident; year 2010)

Although not stated explicitly, it seemed that family members’ preference to have the delivery at home either due to lack of money or for some other reasons might have led to the women to not seek a health facility for delivery.

### Transportation-related delay

As mentioned earlier, because of better health infrastructure, good roads and transportation facilities in Chandigarh, only rarely participants complained about delay in getting a vehicle to reach the health care facility. As a person said:

*Delivery occurred at 11 am*. *I went for hiring an auto rickshaw and at 12*:*30 pm I got an auto rickshaw and immediately took her to tertiary hospital*. *Doctor checked her and told us that she was brought dead*. *She died on her way to tertiary care hospital*. *(*Notes by the interviewer, Maternal Death ID: 16; died of postpartum haemorrhage; Age-32; multigravida (4); urban slum resident; year 2009)

Seven (10.3%) deaths happened on the way to the first point of contact with a health facility or when the women were moved/transferred from a lower health facility to a higher-level facility. It is not clear whether transportation delay (i.e., getting an ambulance in a timely manner) could have contributed to death in the latter case.

### Gap in birth preparedness

A majority of the relatives who were interviewed did not report any birth preparedness; only about 3% (3/68) reported that they were aware of the signs when they have to report to appropriate health facility.

### Seeking care from multiple health care facilities

The perceived level of seriousness of the complications had an impact on seeking care from private health facilities. A few cases narrated they themselves decided to take care in private/higher level health facilities, when they were not getting relief from lower level/public health facility. Among these cases, we also noted that patient doctor communication was poor. Another situation reported was when prior to taking care in the public health care facilities, they had consulted two or more private doctors.

### Health care system level factors

Nearly 88% of the 68 deaths happened in the health facilities. We found evidence for possible health care system level issues that could have contributed to maternal deaths.

Multiple referrals from lower level health facility to higher level health facility happened in one-fifth of the cases, and at least one referral was made in another two-fifths. Severity was reported the major reason for referral to higher-level health facility. However, even a higher-level facility such as a secondary level district hospital, with otherwise all the necessary life-saving equipment, human resources and infrastructure, too had referred the patients to another higher-level facility such as a tertiary care hospital.

#### Delay in initiating treatment and absence of doctors at the time of arrival

Delay in treatment initiation was reported in 10 of the 68 cases. Among all these cases at least 45 minutes had taken for initiating first level of care. Treatment delayed due to absence of a doctor was reported in a few cases. Also, relatives opined that during onsite treatment when they felt that the patients’ condition was severe they did not receive prompt care.

*One hour after delivery there was severe abdominal pain and dai suggested us to take her to the doctor*. *We took her to nearby medical college (tertiary care hospital)*. *When we reached there*, *no doctor was present*, *and for next 2 hours no doctor examined her*. *Doctors were consoling us and the patient died*. *This was doctor’s carelessness*. (Notes by the interviewer, Maternal Death ID: 13; died of postpartum haemorrhage; Age-33; multigravida (5); urban slum resident; 2009)

In some cases (n = 2), caregivers reported that the lack of availability of senior/specialist doctors at the time of medical emergency as a key reason for the death.

*“She was 9-month pregnant*. *Pain started four days before death*. *She was checked in dispensary and medicines were given*. *Pain subsided*. *After that again pain started at 10 pm*. *At 10*.*30 pm we went to a secondary care hospital and she was taken to the labour room*. *There they kept her for one-and-half hour and told us that baby’s heart beat is missing and referred her to a referral hospital (tertiary care hospital)*. *We reached the referral hospital at 1*.*30am & she was immediately admitted and taken to labour room*. *Blood tests were conducted*. *We told them that if operation is needed then do it but they did not do any operation*. *They asked for blood but didn’t transfuse*. *On the second day at 2 o’clock they asked to send one female attendant and we send her sister (women’s sister)*. *She went inside and they said patient’s heartbeat is missing*. *Senior doctor was not there*. *We reached there by morning 1*.*30 am & they didn’t perform any operation till 2 pm*. (Notes by the interviewer, Maternal Death ID: 33; died of other complication of labour and delivery; Age-20; primigravida; rural resident; 2012)

#### Lack of adequate infrastructure or equipment

In a few cases (n = 4), lack of sufficient number of necessary equipment had apparently led to death. For example, one patient was referred from a lower level health facility due to non-availability of enough number of ventilators in that health facility to a higher-level facility. Other patients were already using the existing ventilators.

*“Doctor told us that her heart beat is low and she needs ventilator for survival but we do not have a vacant ventilator*. *Patient died at 11 at night*.*”* (Notes by the interviewer, Maternal Death ID: 57; died of maternal infection; Age-28; multigravida (2); urban slum resident; 2013)

Another caregiver narrated that when they took the woman for delivery at a lower health care facility they were told to come on the next day, as there was no bed available in the labour room; the same incident being repeated on the second day as well. On the third day, the delivery happened at home with the help of a neighbour. But she was admitted in a hospital on the next day for profuse bleeding and she died in a few days. It is not clear, however, whether an institutional delivery might have prevented this death.

### Care pathway analysis

**Fig 2:**
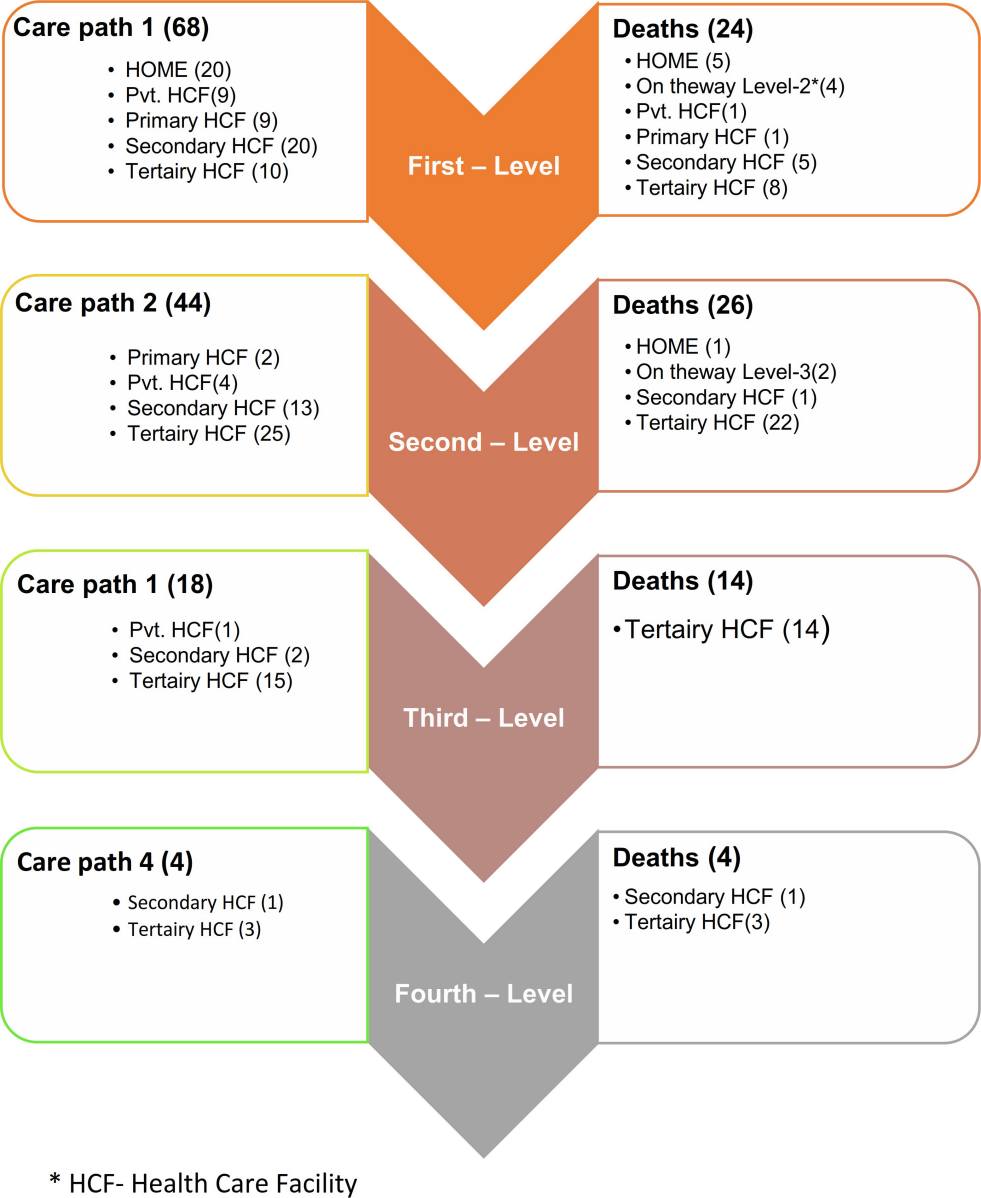
Points-of-care of the deceased pregnant women and number of deaths, point-of-care wise.

Women went through different pathways of care and their points of contact with the health care system varied accordingly. For example, some women were first provided care at home itself by a traditional birth attendant, for some other women the first point of care was at the tertiary health care level. Accordingly, the points of care and four levels of care were presented in Fig 2. About one-third (29.4%) pregnant women’s first point of care was at home with the traditional birth attendant (20/68), while another equal percentage had received care at the secondary level health care facility. About 13% pregnant women had received care from private clinics or primary level health care facility, and only 15% had tertiary level facility as their first point of care. Out of the 68 cases, 24 (35.3%) deaths were reported at the first level care, and 21% were reported at home. About two-thirds (44/68) had two points of care. More than 50% were referred to tertiary level and less than one-third had gone to secondary level of health care facility as second point of care. A total of 26 deaths happened at this level, majority in the tertiary care hospital. One-fourth (18/68) had three points of care, and 6% (4/68) had four points of care (Fig 2).

**Fig 3:**
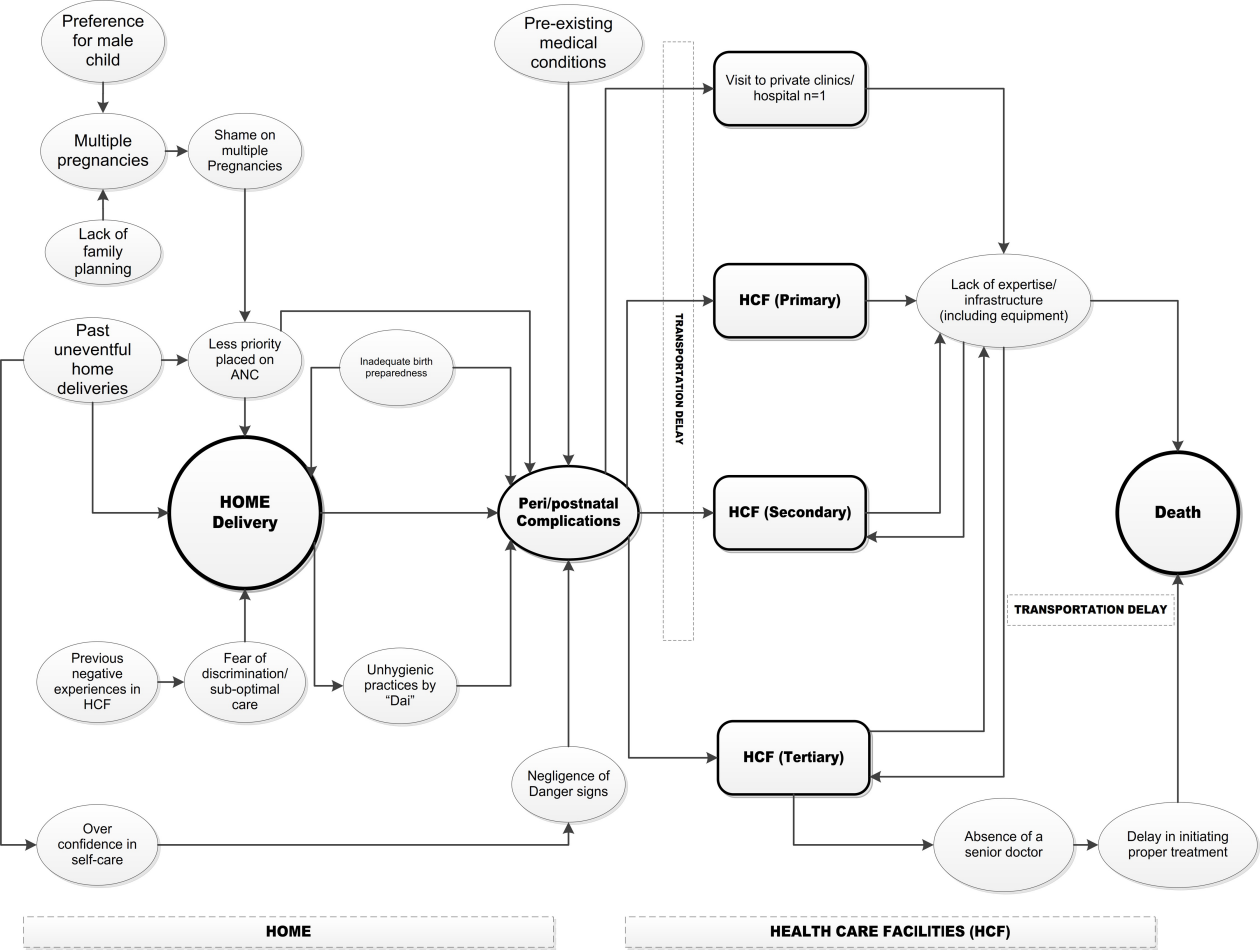
Causes of maternal deaths as perceived by the caregivers of the deceased pregnant women.

The factors identified from the narratives of caregivers of deceased pregnant women, that could have led to the maternal deaths, are summarized in Fig 3. On the left side, the diagram shows the underlying reasons for home delivery and the resulting complications that led to deaths; and on the right side the diagram captures the issues in health care facilities (private and government, and from primary to tertiary care levels) that could have contributed to maternal deaths.

## Discussion

Multiple causes of deaths of pregnant women and young mothers have been reported in the literature including lack of care during pregnancy, natal and postnatal period, or due to lack of resources in terms of manpower, infrastructure, drugs and logistics or financial constraints [11]. It is also emphasized that maternal care needs to be available at the health system, community and household levels. While most studies have focused on medical causes of maternal deaths decided by the medical practitioners, we are presenting caregivers’ perspective on causes of maternal deaths to explain social causes along with medical in this study. We started our analysis with the question whether these deaths were preventable, and we used hybrid models for analysis and presentation, including GIS-based analysis. Use of a combination of delay and care pathway models is a methodological innovation in this analysis and presentation of findings.

All the deaths in the present study were among women who resided either permanently or temporarily in the city of Chandigarh, a city that have adequate primary, secondary and tertiary hospitals [19]. Most of the mothers in this study were from slums and rural areas. Like other studies, maternal deaths occurred more in families living in slums and rural areas than from urban area [24].

It seems ironical that despite having such good health infrastructure, about one third deaths of the women happened at home reflecting not only the higher risk of maternal mortality among women who delivers at home, which is also documented earlier, but also that there is an accessibility issue of existing maternal health services in the study area. The exploration into the reasons that why so many women delivered at home were many, but mostly because of their previous negative experiences at the hospital, fear of approaching health service provider, shame of multiple pregnancy, and overconfidence due to successful previous home deliveries. While poor say in the family, lack of transportation, traditions and religious values had been the reasons for home deliveries in other Asian countries like Bangladesh [31]. The findings of the present study indicate that more than community norms and values, there is felt discomfort in approaching the service provider and discrimination with the women having more children. The social causes expressed in the present study indicate that there is a need to improve the health infrastructure and the behaviour of service provider.

Further, most of the earlier studies have indicated the age of pregnancy, multipara pregnancies as factors that increases the risk of maternal death. In addition to these factors, this study adds the evidence for psychosocial reasons for maternal deaths wherein women were ashamed of approaching health services because of number of pregnancies. The psychological pressure that most pregnant women go through to have a male child leads to multiple pregnancies, which in turn result in shame and guilt. While society is modernising and there are efforts to promote girl child births in India, the health systems need to be more user friendly. The policies, programs and behaviours need to change so that the women are not blamed overtly or covertly for having more children [32]. There is equal responsibility of men for such situation in woman’s life, who are somehow being ignored at the maternal health program implementation level. Also, woman had to deal with various societal pressures like to have son, which should also be dealt in an integrated manner with maternal health programs.

The medical causes identified in our study was similar to the causes identified by various other studies and Sample Registration System Bulletin, India [11]. Postpartum haemorrhage and complications related to puerperium were the major causes leading to deaths of women. Most of these deaths seemed to be preventable. But women died and majority of the women were from lower socio-economic group, from slums and rural areas, migrants and having very little social support. They are also the group who have limited food supply and high burden of anemia, which makes them more vulnerable to post-partum haemorrhage [33]. This profile also indicates lack of availability of information, transport, discrimination, language barriers, and all those associated factors that often push them away from the health services as have been reported in other studies [21].

We identified four levels of points-of-care (See Fig 2). Usually, in each level, more and more women were referred from the lower level health facilities to tertiary hospitals, apparently indicating the severity of the maternal condition. Although this heightened severity in the maternal condition could possibly explain the higher number of deaths reported in the tertiary hospitals (~75%), one would expect lesser deaths in tertiary hospitals given the highly skilled medical personnel and better medical equipment that were supposed to be available in such facilities. However, caregivers of deceased women reported possible delays in tertiary hospitals, and such delays have been reported in other studies as well [14, 18]. Other reason could be delayed referral from lower level health facilities to the tertiary care hospitals, or already overburdened staff and resources in the tertiary care hospital.

The health system factors leading to deaths of mothers have started catching the attention of researchers. However, there are large gaps in the knowledge about the factors for the apparently insensitive behaviour of health service provider. The multiple referrals indicate that there is shortage of equipment and human resource at the institutions. The health facility survey reports from various states including Chandigarh have been reporting shortages from time to time [32]. Another problem that has been identified in Chandigarh is the poor availability of referral vehicle [34]. This study did not explore the reasons for the same.

The present study indicates that the care pathways are determined by the socio-economic conditions in which the women lives and the information available with the women and family about the pre-delivery medical conditions or health conditions of the women at the time of delivery. However, this needs to be explored through in-depth interviews with the service providers and using case study techniques of data collection. Moreover, this study could not capture the near miss cases that can lead to understanding of the gaps that exist in the care pathway.

The delays at home and at health facility have causes which were not in control of the women who have died. The states with more equitable (having low rural, urban and socioeconomic differentials) and less discriminatory behaviours towards migrant, labourers have less maternal deaths as compared to other states. Most of the home deliveries and deaths had occurred between 2007 to 2008. To increase the institutional delivery rate and prevent maternal deaths, Government of India has launched various maternal health interventions under National Rural Health Mission during 2005, like *Janani Suraksha Yojana* [35] to provide financial incentives to pregnant women for institutional delivery, and *Janani Shishu Surakha Karayakaram* to provide free antenatal, postnatal services and sick infant treatment in the government hospitals including diagnostics and provision of essential medicines in 2011. However, the *Janani Suraksha Yojana* scheme did increase the institutional deliveries but uptake of benefits of the scheme was poor due to sub-optimal incentives, delayed payment, problem in arranging for residence proof especially for the high risk vulnerable population and lot administrative work in fund disbursement [36]. Absence of birth preparedness, community link worker and lack of 24/7 availability of referral vehicles in the, especially observed in the, first half of the study analysis period (2007–2015) might have indirectly led to delays and deaths. Therefore, the policy and its implementation is a key to reducing maternal deaths and can help in reducing influence of social causes.

A majority of the maternal deaths were reported from the slum areas, with a high proportion of deceased women from these areas delivering at home, and having allegedly having preference for son or who had to face the social pressure to have a son. Hence, it is recommended that activities to reduce maternal mortality be given priority to those disadvantaged communities living in slum areas. Under the National Urban Health Mission, there are Accredited Social Health Activist (ASHA) in slums who can help increasing awareness among pregnant women regarding birth preparedness and healthy pregnancy [37]. This study provides three clear implementable recommendations: 1) policies are needed to reduce delays and facilitate care pathways that lead to life for all women equally, 2) antenatal care visits should be used as an opportunity to understand the social causes operating at individual, family and community level and providing counselling for women to cope with them, and 3) primary delivery points need to be strengthened (with adequate infrastructure and human resources) to reduce referrals to higher facilities.

## Supporting information

S1 File Quantitative data of maternal deaths reported between 2007 and 2015 in Chandigarh, India. (SAV)

S2 File Qualitative data of maternal deaths reported between 2007 and 2015 in Chandigarh, India. (DOCX)
