# Supplementary material for: Contribution of social factors to maternal deaths in urban India: Use of care pathway and delay models
Source: PLoS One. 2018 Oct 9;13(10):e0203209. doi: 10.1371/journal.pone.0203209 (PMC6177129; doi:10.1371/journal.pone.0203209)
Supplement: S2 File — (DOCX) [file pone.0203209.s002.docx]

**Qualitative narratives**

**UID 1**

Her delivery was done at home. On XX date, boy was delivered at 4 in the evening. Dai came for delivery from village. Baby boy was born, and everything was fine. On second day she complains about having pain in legs. We brought medicine from doctor in dispensary, but this time she didn't get relief, so we took her to WW (secondary care) hospital. After taking her to WW hospital they said patient is dead. She died on the way.  We bring her back home. Pregnancy card was prepared from civil dispensary. Checkup was also conducted there.

**UID 2**

Her delivery occurred on XX date at home. Her card (MCH card was prepared from civil dispensary. She used to go for check up there only. On XX date, delivery was conducted by Dai (Traditional Birth Attendant - TBA) at home only. Boy was delivered normally. 5-6 days after delivery of boy she got fever, then we called private doctor to see her, he gave medicine.  Before the birth of baby also she has been to a private doctor for checkup, doctor told us that he had given her an injection for inducing labor pain so that delivery can occur early. After delivery tetanus injection was also given. 6 days after delivery when didn't get any relief from doctor another private doctor was also consulted. When she didn't get any relief, mean while she also got diarrhea we consulted another private doctor. They told us to ask us to go to either WW (secondary care) or ZZ (tertiary care) hospital. We took her to WW (secondary care) hospital nearby and they started glucose. She was there for 1 day and 1 night after that they sent us to ZZ (tertiary care) hospital. She was admitted there for 3 days and died on XX date at 8 o'clock. Doctor at ZZ hospital told that she died of septic.  She took injections and consumed multivitamin tablets which she got from dispensary.

**UID 3**

Nine months of pregnancy was there. Card was prepared from civil dispensary. Injections were given on time and also consumed multivitamin tablets. 2 injections were given. When she was 4 months pregnant, doctor told us that she has diabetes and then card was prepared from a ZZ (tertiary care) hospital. Doctor started medicine for diabetes. Everything was fine. We were taking treatment from there and we reached there on the date given by doctors. She was admitted on XX date. Baby was born on XX date. When the girl born she got fever and cough. Everything was fine fever used to occur in the morning and evening. She died on XX date.

**UID 4**

She had deficiency of blood in the 7th month. Hb was 5.6. She was taken to WW (secondary care) hospital. She was referred to ZZ (tertiary care) hospital in the city. There they admitted her and transfused one bottle of blood and also an injection was given. Her arm got swollen. Two injections were injected in one bottle. Injection was 250 gm. after that her arm started swelling. We informed doctor that her swelling is not subsiding, but they did not bother. She had cough also during 8th month of her pregnancy. She falls due to rain. We came to hospital and they took her to labor room after that only her dead body came out from labor room. She had delivered still birth and was female. Baby was delivered through normal delivery. she was kept on ventilator for 8 days.

**UID 5**

Heart problem was there 3-4 years back and she used to take medicines. Operation was done from ABC city. She was admitted on XX date. Ultrasound was done 2-3 times to check. Everything was normal. Ultrasound was done before child birth. One and half hour after delivery she died. This is not known what the problem was. She was unconscious for 1 hour.  We were not told what happened to her.

**UID 6**

Attendant told us that mother was not having illness till 9 months. He told us that that he took her to a civil hospital in an ABC city, and also took ANC care there in last month.  She was admitted in the hospital and doctors said that baby died in uterus, then doctors referred patient to a WW (secondary care) hospital. When we reached there the consultant doctors said we are referring the patient to ZZ (tertiary care) hospital in the city. Doctors said she had twins’ baby but died in uterus and there is very high risk for mother in this condition.

**UID 7**

After delivery all the family members were there and were talking with patient. When she was talking suddenly her jaws locked. After that when her jaws were opened till that time she died. After delivery she complained of abdominal pain to her husband, immediately after her complained, her jaws locked. The person tried to open her jaw but till then she was dead.

**UID 8**

She was absolutely normal during pregnancy. She used to go for checkup at a holiday home. Everything was normal in ultrasound. 12 hours before the birth of baby labor pain started at 3 am in night, but she didn't tell us. Next day at 3 o'clock baby was delivered. Dai (TBA) was called at home. 2-3 hours after delivery suddenly pain started. We took her to a ZZ (tertiary care) hospital; their doctors checked her and said she is dead. We also don't know how she died.

**UID 9**

Baby was born at 7 months.  Baby had already died in uterus but there was no complication in delivery. After delivery her color has changed. She looked yellow colored, then doctor was called

**UID 10**

Baby was born at home. Next day at 10 am doctors told she has chest pains and low BP. Then we took her to a WW (secondary care) hospital. Doctors referred the patient to ZZ (tertiary care) hospital and they took her to labor room and checked. Doctors didn't tell any cause of death.

**UID 11**

It was 9 month and 4 days pregnancy. Dai (TBA) was called when she started complaining about labor pain. Dai suggested us to take her to hospital as baby had stopped movement. Initially we took her to WW (secondary care) hospital and doctor checked her and told us that she has low Hb. We can't save her and referred her to ZZ (tertiary care) hospital. Then we took her to the ZZ hospital and admitted her. We were not allowed to go inside. We don’t know anything about her weakness and anything else.

**UID 12**

Nine months of pregnancy have been completed. During pregnancy she had problem of discharge of ear and 20-25 day before death she had fever and swelling in neck. For medicine we used to take her to secondary care hospital nearby. For some time, she got relived but again problem arises

**UID 13**

Delivery was conducted at home and relatives were present at the time of delivery. Dai was called after delivery. Delivery was done at 3.30 am. At the time of delivery patient didn't have any problem and delivery was normal. Severe bleeding was there after delivery and she became pale. Dai (TBA) was called and Dai (TBA) told that she is okay. 1 hour after delivery there was severe abdominal pain and Dai (TBA) suggested us to take her to the doctor. We took her to a tertiary care hospital. When we reached there, no doctor was present and till 2 hours no doctor examined her. Doctors were consoling us, and the patient died. This was doctor’s carelessness.

**UID 14**

According to her husband: I have 3 kids. 2 kids were delivered normally at home. After that one kid was delivered at a civil hospital. All the kids are female (3 kids). Younger child was one year old. When she got pregnant, we came to know in the second month that she is pregnant. From starting we used to go to civil hospital hospital for consultation. We used to go for regular checkups. Ultrasound was done from there. It was 5 months pregnancy and all the reports of ultrasound were normal. They didn't tell us about any problem. After some days at home she started having pain at 3 am in the morning and around at 6 am we took her to a WW (secondary care) hospital. They didn't check her and send me to OPD.  Pain was severe and from 7am to 8 am we waited for starting of OPD session and pain remained as such. At 8 am OPD opened and we prepared card and then she was sent, to Gynecology. They admitted her, but pain didn’t subside. I request them for taking the baby out by operation, but nobody listened. Pain continued for 2 days. She was in severe pain but didn't lose consciousness. Repeatedly she was saying I’ll not survive, and I should take her from here, but no one listened. Doctor didn't tell me what the problem is. Baby was in uterus and at 3 in the morning they told that she died. After that dead body was taken to police. I didn't ask anybody that why she died. My wife died because of the hospital people, but I can’t do anything. If they had done big operation to take out the baby, my wife would have survived. When ultrasound was done they said there is lack of blood and water.

**UID 15**

Pregnancy was of 9 months. Earlier she has two female children. Both the babies were delivered through C-section for XX year and XX year respectively, in a ZZ (tertiary care) hospital through C-section, because during birth of both children doctor suggested that baby has low pulse rate and they don’t want to take any risk. Third baby was conceived in XX month. There was no problem till 5 months of pregnancy. Doctor had prescribed for USG, USG reports state that position of baby was not normal thus mother was instructed to not to do any work. Mother had problem in sitting at the time of 7th month so family took the mother for checkup to a civil hospital, again USG was done, and doctor told us that the baby was not in normal position and instructed us that if we got any difficulty than we should take the mother directly to WW secondary care hospital. After that we went to a WW hospital and had gone for all the routine checkups. EDD was on XX date. On XX date, HB test was done, and HB was found 9gm. After seeing Hb and USG report she had been admitted in hospital and blood transfusion was also done. Doctor told us that due to some technical problem report was not coming right and asked us to have test from outside. HB was checked on alternative days. Mother usually has low level of Hb. On XX date, again USG was done, and this time report suggested that the baby was in normal position and shifted her to CLR (clean labor room). On XX date surgery was done by doctor at 1:00 pm and patient was shifted to ward. After surgery patient was okay till 8:00 pm but suddenly at 8:00 pm vaginal bleeding started, patient’s mother in law called doctor but no doctor came to check her.  Then at 12:00 at night one doctor came and he gave injection to patient and after receiving injection bleeding got stopped. At 5:00 am again doctor came for checkup and gave her injection after 1 hour continuously. Patient died after that.

**UID 16**

Delivery occurred after completing 9 months. ANC was not taken from anywhere. 9-year-old female child was born in a tertiary care hospital who was born by LS/CS. After that 5-year-old male child was born through normal vaginal route at home. 3rd child was also born at home. There was no problem during 1st month to 8th month period. She was behaving normally on XX date, and even at night she was normal. She called me on XX date, and as labor pain started as I reached the home. Neighbors were gathered there, and delivery was also done by them. Delivery was normal, but she fell unconscious after delivery and was not responding. Delivery occurred at 11:00 am. I went for hiring auto rickshaw and at 12:30 pm i got the autorickshaw and immediately took her to YY (tertiary care) hospital emergency. Doctor checked her and told us that she was brought dead and she died on her way to hospital.

**UID 17**

Delivery occurred in full 9 months at home. Home delivery was conducted on XX date. Dai (TBA) was present at the time of delivery. Delivery occurred around 11am in the morning. On XX date, around 10 pm mild pain started and, in the morning, when pain got severe, some relative was called. Baby was born dead. After delivering baby she was fine for 5 days. On XX date, pain arose around 2 pm. Pain was in the left side of chest.  In morning we took her to private doctor. He gave medication for pain and referred her to district hospital in ABC city. Medicine were given but, no relief. Doctor checked her and referred to a ZZ (tertiary care) hospital. We went to there around 6pm. And she was admitted to emergency OPD from SLR (septic labor room). All the investigations were done and admitted. Doctor didn’t tell us anything and on XX date around 4 am she died.

**UID 18**

Delivery occurred in 8 months and 10 days of pregnancy. Normal home delivery occurred. Delivery was conducted by Dai (TBA).  On XX date, delivery was conducted at around 2.30 pm and male baby was delivered. There was no problem for 8 months and 10 days of period. On XX date, pain started at 10 o’clock. Some people suggested checkup from Dai (TBA). Dai conducted delivery. After delivery severe bleeding occurred and bleeding continued till XX date. On XX date, we took her to a WW (secondary care) hospital. Their doctor gave medication and said there is no problem and after checkup bleeding stopped and mild headache was there. After that she was perfectly fine. On XX date, she had headache. And I took her to private doctor. He said take her to the WW hospital. There she felt giddiness and she had a fall then around 7 pm I took her to the hospital and she was admitted immediately. Doctor said there is no life remained in patient’s body. She is dead. Doctor said because of heart attack and tension she died.

**UID 19**

This was her first pregnancy. She used to go to a WW (secondary care) hospital for checkups. Card was prepared. She had no problem. During starting of 7th month pain started and was severe. Nobody was at home so were not able to go to hospital for checkup. So, a local Dai (TBA) was called and she checked her and told that delivery can be done at home and pressed her. Baby was delivered. Baby was still birth. Dai (TBA) had inserted her hand to clear the placenta without wearing gloves. She was screaming and suddenly half of placenta came out and half remained attached to her. She was saying again and again about pain. But she died.

**UID 20**

According to her husband- 13 years of marriage have been passed. Our 1st baby born after 3 years of our marriage and baby was delivered normally at home.  After that we had 3 babies one born at 8.5 month, other at 7.5 month and 8 months respectively. Out of our three babies one was still birth and 2 were alive. Her eating habits were poor before and after conceiving of baby. She was very weak. I am a laborer and I used to give her medicine before going for work. But she never took medicine after that. Food and medicine used to remain unused as such. From the beginning she used to go for checkup at civil dispensary. Sometime when she got fever then she took medicine from private doctor. This time in 7th month mild pain started at 3o’clock and at 11 in afternoon severe pain got started. Dai was called, and baby was delivered. 30 minutes after delivery baby died. We took baby to cremation ground. After coming back, I went to market to bring something to eat. Then I got a call that she is not well. She was having severe pain in abdomen.  Then we took auto and took her to hospital, near 23-24 lights she got unconscious. But her heart was beating, and her body was warm. We took her to emergency in hurry. But doctors checked and said she is dead. She had a lump on her one ear for that she got operated 2 years back in 16. They told her to take medicine for 1 month, but she left from there in 1 week. After that she was not having any problem.

**UID 21**

According to her husband- 4 years of marriage have been passed. One miscarriage occurred three years after marriage. One year after miscarriage she had conceived again. From the beginning we consulted from WW (secondary care) hospital and local dispensary. All tests were done and were normal. In WW hospital, LFT (liver function tests) was done. 1st test was conducted on XX date and report came on XX date and test was repeated on XX date. In 9 months of pregnancy there was no problem. She was fine till last. There was no problem. On XX date she complained of itching. They admitted her, and ECG was done. and referred to a ZZ (tertiary care) hospital at 3.30 pm, and they told us that her heart seems weak. On XX date some spotting was there. And ECG report was normal. After some time, doctors told us major operation has to be done. We told them that we are ready. We told her that doctor is saying that her heart is weak. Everything was normal till 16 night. Sample was given on 16th night. Report was normal till 2.30. On XX date, baby was born at 11.30. At 12 o’clock they told baby was born. After birth of baby they told us that file was prepared. At 2.30 they told us about tests. Oxygen was given to the baby after birth. Didn’t tell us anything about that. They asked us for injections from outside. We brought the injections and 2 injections were given. At 4 o’clock they told us that she died. We have a complaint that even after knowing that there was a heart problem they didn’t call any heart specialist. Because of this she died. After delivery when her mother in law went inside oxygen was attached.

**UID 22**

Five years had passed since her marriage. Before this baby she has 5-year-old baby who was born normal by surgery. After this baby one girl was born. This girl was also born by operation, but she died. In XX year, she again got pregnant. There was no problem from starting. She used to consult in a WW (secondary care) hospital and there was no problem in reports. She went for checkup on XX. There they admitted her and asked for papers from home. When she was admitted they told us that they have to keep her admitted for 1 week. They kept her admitted for 1 day and then she came back home on XX date. On XX date, at night she went to toilet and some spotting was there. On XX date, at 10.30 pm at night we went to WW hospital and immediately they admitted her and at 2.30 took her to operation theatre. At 3 o’clock girl was born. At 3.15 they asked us to bring all the medicines urgently then asked for arranging blood. At 5.30 they said she is very serious and have to refer her to ZZ (tertiary care) hospital immediately. Then we took her to the other hospital and they admitted her immediately. When the patient came out of operation theatre breathing was given to her via pipe. When we took her to there, she was unconscious. At 9 am hospital people told that she is dead. At ZZ (tertiary care) hospital, people asked for medicines worth rupees 32,000. At 9 am she was dead. ZZ hospital people said she was dead in the WW (secondary care) hospital only. We can only say that they (doctors) had cut down some blood vessels at WW hospital. Because of that only there was so much of bleeding. People (service providers) at WW (secondary care) hospital have killed our daughter in law.

**UID 23**

This was 6 months pregnancy, gravida 4 and the youngest baby was 3.5-year-old. At the time of 2nd baby, consultation was done from a tertiary care hospital. At that time doctor told that she has heart problem and she have to take medicines until she is alive. One year before her death she stopped taking medicines for heart. 4-5 days before her death, she got fever and cough. She bought medicines for cough and fever from the doctor. And she recovered well. On the day of *rakshabandhan* (festival) she became breathless at home. At 12 am we took her to a WW (secondary care) hospital in rickshaw. They referred to a YY (tertiary care) hospital and she died on *rakshabandhan* in the morning.

**UID 24**

Delivery occurred at 8th month. ANC was un-booked and unsupervised. She was suffering from epilepsy since she was 7 year of age. She also had episode of seizures during her pregnancy. She had paralysis of left side. It was her first pregnancy and she was suffering from headache and vomiting throughout her pregnancy. At 8 and half months of pregnancy she went out for taking water in rain and she slipped. She got injury in her head and started paining. she had severe headache and had episode of seizure at that night. On XX date, we took her to a WW (secondary care) hospital, there doctor admitted her for 1 day and referred to another ZZ (tertiary care) hospital in the city next day. She was operated for her brain and also C-section and was discharged after 5 days and doctor told us that there is no chances of survival of this patient and instructed us for taking her to home and referred to another YY (tertiary care) hospital. After taking her there, the doctors referred her back to the ZZ (tertiary care) hospital where surgery was done, then we took her to WW (secondary care) hospital, and their doctor told us to take her to home and we took her to home. For next 8 days she was in coma at home. Food was given through pipe. She died at 2:00 pm on XX date.

**UID 25**

Delivery occurred in complete 9 months at tertiary care hospital and it was C-section. During pregnancy she had pain in lower abdomen from first trimester till delivery, and she had no other problem. ANC was booked (registered) and supervised. 90 IFA tablets and 2 TT injection were taken. EDD was on XX date. On XX date, at 7:00 pm she had severe pain in her lower abdomen and she was immediately taken to the WW (secondary care) hospital by autorickshaw. As soon as we reached she was admitted in hospital. I met the patient at 11:00 pm and there was no sign of bleeding, but pain was not continuous. She was shifted from labor room to other room. On XX date, doctor was taking morning round and bleeding started at that time and she was shifted to labor room immediately. She had low Hb before delivery and doctor suggested for C-section. She was bleeding since 9:00 am in the morning. Delivery occurred at 12:05 in afternoon, it was her first delivery and mother and baby were fine after the delivery. She was shifted to maternity ward and was on blood transfusion and she was all right till XX date. On XX date (1 day after shifting), she was talking to us normally and was on glucose drip and was sleeping at that time. She woke up at 5:00 pm in evening she asked for water and she was complaining pain in her chest and difficulty in breathing. I told the condition to nursing sister and she did not listen and told me that doctor is coming, and she was running here and there to call doctor. In a while 3-4 doctor came and had put oxygen and referred to ZZ (tertiary care) hospital. We took her to the other hospital where doctor told us that we took time to bring patient to hospital and she died within 4 hours after coming to here. Doctor told us that she died due to heart attack

**UID 26**

According to respondent- she had 2 children earlier out of that one was born in hospital and other at home. The youngest child is 8 years old. She had conceived again. Check-up had been done in dispensary twice and once AA city in a big hospital. Iron folic acid and red tablets were consumed, and she also used to consume tablets for (multivitamin tablets) weakness. She came from another state one month back. Ultra sound was done and there was no problem in ultrasound. Pain started on XX date at night. Pain got increased so went to WW (secondary care) at 2 o'clock in night and she was immediately admitted. We got auto immediately and took her inside at 2 o'clock admitted in WW (secondary care). On the 2nd day baby was born at 4pm. We had not seen her from 2am to 4am. Baby was shown to us at 4 am. Baby was not able to breathe.  When I had seen the baby, baby eyes were closed. When I called him, baby opened eyes. I have seen my wife bleeding heavily and urinary catheter was attached. They performed a surgery but didn't tell us. When we saw her eyes were open and they said to take her to ZZ (tertiary care) hospital. Doctor from WW (secondary care) came with us to ZZ (tertiary care) hospital. They said that she is dead and didn't tell any problem and we came back from ZZ (tertiary care) hospital at 7 o’clock and baby also died  after 1 day.

**UID 27**

Delivery occurred in 8th month and it was Normal Vaginal Delivery. Delivery was conducted by Dai at home. Baby died before birth. ANC was booked and supervised. TT received. IFA tablets were not consumed properly. During 1st trimester normally vomiting occurred. There was no complication in 2nd and 3rd trimester. USG was done on XX date. Findings of USG were normal. Doctor said baby descended. We took her to some dispensary. There after checking the position by pressing, then position of baby was normal. On XX date water broke at 7.30am. Dai was called at 8.30am and she said there is no need of taking her to hospital.  Delivery will be done in 10 minutes. Delivery was done at 9.15 am. and a dead baby was born. This was her 1st baby. After delivery we went for cremation of baby. After half an hour they called us and told that severe bleeding has started.  At 10.30 am after coming home her umbilical cord was cut. She was not able to speak, and her one eye was open, and one was closed. She was not able to speak. I went to take auto and it took half an hour to bring auto, we took her to a private nursing home at ABC city, then we called doctor and after checkup doctor immediately referred her to YY (tertiary care) hospital. After reaching there we called doctor. Doctor pressed the heart and she breathed once and then she died.

**UID 28**

Patient went to hospital at 4 am with abdominal pain. A neighbor and relative (her sister in law) told to ANM that she had pills from private clinic and she is 2-3 months pregnant (9 weeks). Gravida 2nd.She died at home. Her house was locked, and everyone has left for their hometown.

**UID 29**

This was her second delivery. Baby born was full term i.e. 9 months. Baby was born by caesarian section, because baby was inverted in mother’s womb. MCH card was prepared from a nearby civil dispensary. 40 IFA tablets were consumed and T1 and T2 were received from Civil dispensary, in Chandigarh. Vaginal bleeding started at 2 am in night. They reached a YY (tertiary care) hospital at 4.30 am and ultrasound was done, and they said caesarian delivery must be done if you want to save the baby. Baby was delivered at 9.30 am. Baby cried and breathed after birth. Baby was born healthy. Her vaginal bleeding didn’t stop so they referred her to a ZZ (tertiary care) hospital at 12 pm. There, doctor suggested to remove her uterus and operation was done and uterus was removed by operation till 2pm. vaginal bleeding didn’t stop after operation. 5 bottles of blood were Transfused and at 11.30pm she died. Total money spend by family was 90,000 by loan and transportation used by family was auto rikshaw from home to first hospital and ambulance from first to second hospital. Mild vaginal bleeding started on XX date, but bleeding become more severe on XX date at 2 am in the morning.

**UID 30**

Landlord of the diseased told that we all were busy in preparation of festival (*janmashtami*). Our tenant’s wife started having pain and she was taken to a WW (secondary care) hospital. After some time, they came back at 10:00 am. At 1:30 pm she felt severe pain again and went hospital. Respondent got call about her death at 5:00 pm on XX date.

**UID 31**

In starting there was no problem. Card was prepared in a WW (secondary care) hospital. All tests were normal. Delivery occurred on full term. Bleeding started in initial days of 9th month and after that water started discharging. Then bleeding had subsided itself at home. This happened earlier also. Her elder son was also born through big operation (C-section). At that time also, uterus’s opening was obstructed. Pain started 2 days before the delivery and we immediately took her to WW (secondary care) hospital. She was admitted there, and we were told that she has low hemoglobin level. They sent us to bring blood. After we came back we saw that baby girl was born. We have seen her after 2 and half hours. She was trying to sit, and then suddenly without telling us anything people referred her to another ZZ (tertiary care) hospital. We took her to the hospital, after reaching there they were compressing her chest and 3 bottles of blood was transfused but still she was unconscious. She died during chest compression. There was no problem in admitting. Death occurred due to surgery in WW (secondary care) hospital.

**UID 32**

Before this baby she has one boy who was delivered normally at home. During her pregnancy she used to go for checkup at a local civil dispensary. Injection and tablets were taken from dispensary. There was some problem in liver during pregnancy. There was infiltration of Water in the liver. On XX date, pain started in the day and delivery occurred at 10 in the night. She was fine till 6 days after delivery. Delivery was conducted at home. After 6 days, suddenly whole body was started swelling. And on the same day pain started in the legs and then we took her to a WW (secondary care) hospital, and they told us about the same liver problem and referred her to another ZZ (tertiary care) hospital in the city, and she was admitted to that hospital, and after 12 hours she died. Tertiary hospital people didn’t tell anything about cause of death.

**UID 33**

This was her first pregnancy. In the beginning card was prepared from local dispensary. Iron-folic acid tablets were taken. TT Injections were given. All tests were normal. There was no problem. She was 9 months pregnant. Pain occurred four days before death. She was checked there, and medicines were given. Pain subsided. After that again pain started at 10 o’clock on XX date. At 10.30 we went to WW (secondary care) hospital, and she was admitted and taken to the labor room. There they kept her for one and half hour and told us that baby’s heart beat is missing and referred her to ZZ (tertiary care) hospital and we reached at 1.30 and she was immediately admitted and taken to labor room. Blood tests were conducted. We told them that if operation is needed then do it, but they did not operated. They asked for blood but didn’t transfuse. On XX date, at 2 o’clock they asked to send one female and we send her sister. She went inside and said here heart beat is missing.  Her sister saw that she is no more. Senior doctor was not there. We went there on XX morning at 1.30 am and they didn’t perform any operation till 2 pm on XX. Didn’t tried for delivery.

**UID 34**

This was her second delivery. She visited a hospital near to home for checkup from the beginning. She used to go to hospital whenever she was called. There was no problem in the pregnancy. Normal delivery was conducted by Dai at home on XX and there was no problem after delivery. After 2 days of delivery she was fine and on XX she had severe back ache.  Dai gave her tablet for pain, but pain did not subside. Then she was taken to on XX we took her to YY (tertiary care) hospital.  On reaching she was immediately admitted. For first 2 days they prescribed medications. She was having pain in back and on the right side of abdomen. Pain did not subside, and tests were conducted. It was told that there is abscess and surgery has to be done and she was operated for pus/abscess on XX, after operation she was fine, and on XX doctor told that she got 2 heart attacks and she died during 3rd heart attack. 2 days after delivery when she had pain then Dai gave her a lot of massage. Because of the massage internal injury was there.

**UID 35**

She already had 2 babies before this baby. she had consulted in a WW (secondary care) hospital from the beginning. Card (maternal and child health card) was prepared from local dispensary. She had no problem during her pregnancy. On XX at 9pm she started having mild pain and she was taken to WW (secondary care) hospital, after reaching hospital she was immediately admitted and on XX date, at 10.53 pm delivery was done through operation. Severe bleeding was there after delivery.  When bleeding didn’t stop they referred her to ZZ (tertiary care) hospital. It was apparent that some internal blood vessel was cut in the surgery that was done in the previous hospital. Then on the same day she was referred to the ZZ (tertiary care) hospital. Bleeding was continuous. Admitted in ZZ (tertiary care) hospital, and surgery was suggested to stop bleeding. Operation was done there on XX but bleeding didn’t stop. And 3-4 hours after surgery she died. Bleeding was continued after death also.

**UID 36**

Earlier also she had difficulty in breathing for four years. During this pregnancy all the tests were done in a WW (secondary care) hospital. Before this pregnancy she has 3 kids. 20, 17 and 15 years old respectively. She got pregnant suddenly. She got to know about her pregnancy in 3rd month. After that card was prepared from dispensary. 2 TT injections were given, and iron folic acid was also consumed. 5 months of pregnancy was completed. On XX severe pain in neck started at 7 pm in the evening, so we were taking her to WW (secondary care) hospital and then suddenly pain subsided, and we came back home without going to the hospital.  When came back home then her neck started paining again. Then immediately we took her to WW (secondary care) hospital, and she was admitted in emergency. After some time, she was taken to the labor room. They gave 2 injections and pain subsided. They kept her in the hospital for some time and send back at 12 o’clock. Then after coming home she had dinner at 2 o’clock at night, she drank tea and after drinking tea she lied down, at that time she was fine. then she fell down and again took her to WW (secondary care) hospital. and she was taken to labor room. There they pressed her chest and told that she is dead. Baby was in uterus and she died.

**UID 37**

Before this child she had 5 more children which were born normal at home in UP. In this pregnancy she had not prepared any card, neither she had taken any tablets nor any injection. She had consulted a private nurse in adjacent city, and her husband was not sure about whether the nurse had given her any injection or not. In the 1st 4 months of pregnancy she was in other state. In the 5th month she came to this city. Mild pain started on XX night. At 12 pm pain got severe. And at 1pm in night we immediately took her to a nearby private nurse. She was admitted in night at 1 pm and at 8 am she had delivered 2 dead babies. One boy and one girl were born. Both babies died in uterus only. After that nurse discharged her at 9 o’clock. Severe bleeding occurred after delivery. Nurse said it will stop on its own and we came back home. After giving injection nurse send back and after coming home she asked for tea and after 10 minutes she died. Bleeding was severe.

**UID 38**

This was her 1st delivery.  MCH card was prepared from a local civil dispensary in 4th month. ANC was booked and supervised. T1 and T2 were received and IFA was consumed. Child born on complete time through normal vaginal delivery. Exactly after 9 days of child birth mother died. Baby born on XX. Water broke on XX at 10.30 pm in night and at 11 pm we reached a WW (secondary care) hospital and baby born at 1pm at night. 1 day after birth of baby got jaundice. She was discharged on XX. After 4 days on XX Sapna started having severe pain in her stomach.  and at 11am we took her to the WW (secondary care) hospital. After conducting test doctor told us that she is suffering from tuberculosis. Again USG scan was taken in at hospital which showed endometrium is thickened measuring 2-3cm in width show heterogenous hyperplastic foci suggestive of retained product of conception which appear infiltrating into anterior myometrium. They took sample of her intestine and from that come to know about tuberculosis. On XX morning her condition worsened then she was referred to ZZ (tertiary care) hospital. All the necessary tests were conducted again, and doctor told us that here infection has increased and her survival is difficult. On XX at 4.45 pm she died at ZZ hospital. Baby was absolutely healthy. And his eating habits are alright. At the time of birth weight of baby was 1.910kg

**UID 39**

This was her 1st pregnancy. Card was prepared from ABC city in the beginning of pregnancy. ABC was her parental home. She came to this city in 2nd month of pregnancy. She was suffering from typhoid fever. She took medication from hospital. During 2nd month, card was prepared from district hospital in a neighboring state. Along with typhoid she was also having pain in stomach. She had taken medicine from a WW (secondary care) hospital for 1 month. But she didn’t get any relief. Pain remained as such. Doctor gave all the medicines but no effect.  On XX date, she was admitted in ZZ (tertiary care) hospital. They told that there is some internal problem of blood vessels, operation has to be done. Then they operated her baby was normal and after surgery also baby was normal. On XX date, operation was done she became more serious and she died. When she died baby was in uterus.

**UID 40**

This was her 2nd pregnancy. Previously abortion occurred in 4th month. This time from starting she consulted in dispensary. Sometimes stomach ache used to occur. Iron folic acid tablets were also taken. All tests were conducted no visible problem was there. On XX date, she was fine. On XX pain started at 9 O’clock then we took her to a private nursing home in the city.  Immediately took her in tempo. In 20 minutes, we reached there. There they checked and referred to a WW (secondary care) hospital.  On reaching there they admitted immediately. They told that blood is less and asked to give her blood. Along with pain bleeding started in the morning. Till 12.20 kept in 16 and then referred to ZZ (tertiary care) hospital. Reached hospital at 12.45. When reached there it took 30-45 minutes to prepare card till then she lied as such.  After preparing admit card they admitted and said child will not survive and they got our signatures. and at 2.30 she died. Baby was unborn. Doctors tried hard to stop bleeding, but bleeding didn’t stop and while bleeding she died. According to sister in-law of - if after reaching the hospital if they give immediate treatment, she would have survived. Because it took 30-45 minutes to bring the file and during that time no treatment was given.

**UID 41**

This was her 2nd delivery. Baby born on full time after completing 9 months and delivered by CUP. On XX, 8 am in the morning water broke and we took her to WW (secondary care) hospital and admitted immediately. ANC was booked IFA consumed and T1 and T2 were received from com in 4th month. On XX, 8.30 in the evening tablet were given to induce artificial pain through the urinary route and pain started in the morning. On XX, 11 am morning boy was born. Baby was absolutely normal and at the time of birth weight of baby was 3.75kg. 1 hour after the delivery, mother died. Doctor said due CUP induced delivery infection occurred because of that she died. She age was 30.

**UID 42**

This pregnancy was her 5^th^ pregnancy. 1st daughter is 5-year-old. Then one was 6-month abortion. In last, complete 9-month pregnancy, live born son died and one 8-year-old son is alive. Her in-laws used to live in other state. Her parents lived in a village in this city, that’s why she was living here with her husband in rented house for past 2 years. Her mother told that for 4 months she didn’t tell anybody that she is carrying a baby.  Then because of her changing activities when I asked her forcefully then she told me that she is 4 months pregnant. But she didn’t prepare card from anywhere. Neither she consulted anywhere in private. Even after our advice she didn’t consulted. Otherwise she was fine and worked well. She used to eat well. Few days before the child birth she and her husband had a fight with us and he didn’t let her come to our house. According to us the child born on complete time. 2 days before delivery she had minor pains. But she didn’t consult anywhere.  Then on XX at 3 o’clock we came to know that she gave birth to a daughter. Her room was 3-4 room away from our room. Our sister in-law’s daughter told us. We thought that the delivery occurred before 2 o’clock. It might be possible that delivery took place even before this. We came to know at 3 o’clock and went to her immediately. When we saw her, baby was lying in front of her. Her placenta was inside only and at that time baby was attached with placenta. No one dissected the umbilical cord. Her stomach was like stone. We called an autorickshaw immediately and within 15 minutes we took her to a civil hospital (first referral unit/primary care facility). When we reached there, hospital people cut the umbilical cord in the autorickshaw and took her inside on structure. Inside the hospital they removed placenta and gave her 2-3 injection and she slept. Then she asked for something to eat then we brought tea and biscuit to her. But she didn’t drink tea and started shouting that there is pain in my chest. When we saw her at 3 o’clock when we saw her legs and arms were swelled along with that her stomach turned like stone. But she was not bleeding much. When she complained of pain 15 minutes before her death then we called a nurse, she started checking and after coming out told us that she is dead. When we saw her green foam was coming out of her mouth and nose and her arms and legs were swelled. She died at 5 o’clock.

**UID 43**

Any relative of her, who was along with her at the time of disease or death was not available. Her husband left the place after her death. Her aunt who was present at the time of death had also left to the other state. And this respondent is her aunt’s mother in law who was not present at the time of death, but she told us whatever she heard of. This was her 1st pregnancy. She got her card prepared from local dispensary. Once she went to consult a doctor in a WW (secondary care) hospital. She had already decided that she will be having delivery at home as she was scared of hospitals.  Her husband and other relatives tried to convince her for institutional delivery, but her reply was adamant about home delivery. She was having swelling in legs from the beginning. Rest her eating habits and daily work routine was okay. On the morning of XX she started complaining about pain. Pain continued on XX as well. On XX dai came and her delivery was done on XX at 4 o'clock in the evening. She was also suffering from fever 3 days before the delivery and for which medicines were taken from a private clinic. She was fine after delivery. In the evening she ate dal with bread but vomited, then she lied down then she wake up at 9 o'clock and ate *roti and daal* and she told that her stomach is aching severely. Pain increased so same night we took her to a YY (tertiary care) hospital.  She was admitted there for some time then blood started coming out of her mouth. She died at 1 o'clock in night. Large amount of blood came out of her mouth that night.

**UID 44**

This was her second delivery. From the beginning she used to go to local dispensary for checkup and she got her card prepared in civil hospital (first referral unit). There was no prescription of iron folic acid tablets on card, she was not prescribed iron folic acid tablets either from outside or from local dispensary. On XX she started having pain at 7 o’clock and we took her to civil hospital (first referral unit). On reaching there they told us to come tomorrow. Pain was mild. Next day i.e. on XX in the morning we took her to dispensary, but they again told us that baby will not delivered right now and told us to come next day. On XX we took her to dispensary and they said she is not going to deliver baby today. We came home and after coming home on the next day all of us went out for our work. Nobody was at home but when she was having pain since morning. She was having pain whole day on XX and in the evening with the help of neighbor her delivery was conducted at home. All of us came back in the evening. She was absolutely fine for 2 days. Then on the 3rd day suddenly she started having pain in abdomen.  We took her to a WW (secondary care) hospital, but they referred her to YY (tertiary care) hospital we went to that hospital and they said it is septic. Surgery has to be done. On the 2nd day she was referred to another ZZ (tertiary care) hospital.  We went there, and she was immediately admitted. Her mouth got swollen. 2 bottles of blood were transfused. A machine was attached to her neck. No senior doctor was available.  She remained in same condition for 2 nights in at the hospital and she died. She was also kept in ICU in between.

**UID 45**

This was her 3rd pregnancy. She had 2 kids, one 11-year-old boy and one 9-year-old girl.  We didn’t want the baby, but by then she had conceived. Before 6^th^ month of her pregnancy she used to live in the other state. In the beginning of 6th month ultrasound was done, and then she came to know about pregnancy. Card was prepared in 6th month in a civil hospital, and after that 2 injections were given, and iron folic acid tablets were started. Then onwards regular checkups were conducted. Baby was born at complete 9 months on XX at 10 am. Before delivery discharge of water started at 7.40am. Labor pain started at 8 am. Delivery was conducted by Dai (TBA) at home. After one hour of delivery her head started paining and she was taken to civil hospital immediately. She came home on XX. She was fine that night but in XX morning she had pain in chest and head. She was again taken to ESI. She was admitted there for 1 day and 1 night. At night she had difficulty in breathing. Then she had referred her to a WW (secondary care) hospital. On XX she was admitted to there on the same day she was referred to ZZ (tertiary care) hospital as her headache did not subside and chest pain also remained as such. We immediately came to hospital and admitted her, but they didn’t tell anything. At 5:30 she died. When she was brought there she had difficulty in breathing. In all this no one cared about for the baby. At 4.30 at WW (secondary care) hospital baby didn’t breathe. Baby was taken to emergency and checked there, and we were told that baby has got pneumonia and baby died then and there. Baby was normal looking, and no visible problem was there. No treatment was given to the baby.

**UID 46**

This was her 1st pregnancy. In starting ANC card was prepared from XX and one TT injection was also taken from ABC city. From 6th month card was also prepared from local civil dispensary. Then onwards she got her regular checkups from there. Consumed as much iron folic acid tablets as provided. Tests were conducted. Everything came out normal. Otherwise she was normal. We said we’ll conduct delivery at hospital but previously she said she will undergo a home delivery. Pain started at 9pm on XX. EDD was on XX. At the same night pain started, dai (TBA) was informed and baby was delivered at 10pm at home. immediately after the baby was delivered she asked what baby she delivered. Then she asked for water. I touched her mouth with wet finger. Then gave her a little milk. After 30minutes of delivery she got unconscious. Immediately took her to a civil hospital. There they didn’t check and said take her to WW (secondary care) hospital. Then admitted her to emergency and take her inside. After that didn’t show us. She had no bleeding. Unconscious of that time she didn’t regain her consciousness. They did not tell us any cause of death.

**UID 47**

This was her 3rd pregnancy. Elder daughter is 14-year-old and 2nd is 12 years old. This time it was 3rd month of pregnancy and she got her card from a nearby civil hospital. Iron folic acid tablets were received. Tablets were consumed thrice because consuming those tablets induced vomiting. 2 injections of TT were given. She had a thought that her delivery should be conducted in private hospital. Whenever the health workers of civil hospital used to visit her they used to advise her that you should get her delivery in government hospital. But she refused to take that advice.  Because of this many time there were arguments in their house. Sunita had gone for ultrasound twice. She showed the 1st report to civil hospital doctor. Then that doctor told her to redo the ultrasound. Then she undergone for ultrasound on XX and showed the report which was normal. She had asthma 2 years back. Sometimes she used to get breathing problem. For that she used to take medicine from private doctor. On the basis of ultrasound EDD was from 23rd to 26th January. But there were no pains till 26th January. Then a local doctor told us about a private hospital. Then we went there on XX at 9 in the morning. There when they saw the EDD they said time has exceeded. So, we should admit. Then they said she has problem of asthma and this pain will not happen. She has to undergo operation. On XX we went to that private hospital at 10 am and operation was done at 2.30pm. After operation baby was fine and she was also absolutely fine. At 3.30 heavy bleeding started. With time bleeding increased. Bleeding was so much that blood started coming out of her big operation’s stitches. Because of her asthma problem oxygen was also given. When the bleeding didn’t stop they told us to deposit the money and said we are calling senior doctor from outside. At 7 o clock we deposited the money and at 8 pm another operation was conducted, and her uterus was removed. After 2nd operation she was normal till 12 o’clock in the night. Then suddenly she had a seizure. During the seizure her tongue turned blue and her foams come out of her mouth and blood was also coming out of her mouth. Then they said she become more serious. They send her to other hospital. In 10-15 minute we took her to another hospital. They examined and said she died long ago. You brought dead body here. (Her baby was fine, and they had an expenditure of 70,000)

**UID 48**

Before this baby, she had 2 girls and 1 boy which were delivered normal at home. This time card was prepared in 2nd-3rd months from local dispensary. Iron folic acid tablets were received but not consumed. The blood investigations which they had done showed less blood, rest was normal. 2 TT injections were given. one ultrasound was done. LMP was on XX and EDD on XX date. Pain was felt on XX morning and the neighborhood dai was called and in 2 hours normal delivery was done. baby was also normal after birth and the mother was also normal. From her seventh month of pregnancy pain started in her one molar tooth. she used to take medication from a private doctor. Slowly so much of pus was formed in that molar that along with that pus lots of swelling used to come. left side was always swollen. Her pus was drained many times, but she was not cured completely. on the 3rd day of delivery i.e. on XX date at 9 in the morning she vomited and at that time head started aching too much and stomach also started aching too much and left side of her face also started twisting. Then we thought she is suffering through hemiparalysis. We took her to village. In village a doctor who treat this checked her without any treatment referred to a district (secondary care) hospital in the neighboring state. We reached there by 2pm, there they 1st took her to the emergency and gave her 2 injections to stop pain but pain didn't stop. Her headache and stomach pain remained as such. Then we took her to female ward there they inserted hand inside her and took out some waste material and then her stomach pain subsided. Then her parents said it is not right to keep her here and we should take her to private hospital. We took her too private hospital nearby. There they checked her and immediately send her to YY (tertiary care) hospital. That evening only, we took her to that hospitals. They took her in emergency ward there they said x-ray of her head has to be taken. At that time, she was not moving her hands and legs. She was calling her mother over and again. Then one injection was given which didn't anesthetize her then another injection was given, and x-ray of her head was done. After doing the x-ray no injection was given and no treatment was prescribed. She laid as such. Staff-there told that treatment will start when senior doctor arrives. On the same night milk was given to her with spoon and she drank it. Otherwise she was unconscious. Then on XX foam was coming out of her mouth. Then with a great difficulty we searched a doctor when he checked she was unconscious and pump was attached. that day she was remained on the pump, but she didn't regain consciousness. On XX one bottle of blood was also given. Breathing was given with pump and 2 bottles of water were given. On XX suddenly she started breathing with difficulty. On the same evening she was finished. Her tooth abscess and swelling remained as such.

**UID 49**

Before this baby she had 3 more children, two girls and one boy. During 4th month she had her antenatal checkup and card made from local civil dispensary. She used to go after 15-20 days. Blood tests were also done, injections were also given, and tablets were also consumed. She did not have any problem she was fine and used to eat and work properly. On XX date, she went to field and she fainted there, and bleeding started. No one was at home, but neighbor help her and took her to home and called her husband. Her husband came around 12:00 and at 12:30 she was taken to tertiary care hospital she was bleeding, but no pain was there. She was admitted in and told that deficiency of blood and asked to bring blood. Two bottles of blood were transfused and in evening pain started at 7:30 pm and she was referred to WW (secondary care) hospital. She was taken by hospital vehicle. She was admitted immediately and checked and were informed that baby died inside, and to save mother they must do operation. Earlier all the baby was delivered normally but this time they were doing operation and did not informed us. Same night operation was done, and baby was delivered baby was still birth. Then was shifted to ward. When her husband went to meet her she was fine, but she was bleeding severely. Hospital people transfused blood but all in vain, as she was severely bleeding. Third day they told that X-ray has to be done and after doing X-ray they told us that she has to undergo operation again. And operation was done; we were not informed about the problem. She was unconscious after operation and oxygen was given and put on ventilator she did not opened her eyes. Next day she died.

**UID 50**

8^th^ month of pregnancy was there. This was her first pregnancy. Card was prepared from a civil dispensary. Checkups were done every month. Two injection of TT were given. Multivitamin tablets were also given. The dispensary people told us that both mother and baby were fine. But suddenly on XX date pain started in her stomach so we took her to civil hospital. Glucose was injected, and we were sent to home in evening. At night around 2 am pain she again started complaining about pain and she was taken to dispensary from where we were referred to a WW (secondary care) hospital. There at 3:00 am doctor told us that she had some respiratory problem (problem in wind pipe). Doctor told that we can only save either mother or baby. They told us that mother has to be operated and baby was to be kept in machines. Hospital people told us that we have only one machines, and a baby was already kept there, and u should take her to ZZ (tertiary care) hospital. Baby had problem in breathing as her wind pipe was obstructed, along with this they told us there is deficiency of water in mother stomach, so her operation has to be done. Doctor had brought all the material for operation but was not able to operate as mother had low heart rate and in evening at 5:30 both mother and baby died.

**UID 51**

On XX date, when she was brought to ZZ (tertiary care) hospital she had brain hemorrhage. Her brain was completely dead as told by doctor, but other parts of her body were functioning. She was on ventilator. She was kept under observation. She was treated for 5 days, after five days doctor told us that other body parts were not functioning. Her husband told us that because of fluids her body started rotten, but doctor told us that now we cannot say whether she is dead or not because some of her body parts were still working. But her husband did not listen to any one and brought her body from hospital on XX and cremated on. XX date.

**UID 52**

This was her second delivery. She was 30 years old. Card was prepared in civil hospital (first referral unit). Her ANC was registered in government civil dispensary. She was given injection and tablets there. She had previous history of abortion 8 years ago. She had one girl child who is 10 years old. Her LMP was XX and EDD was XX. On XX she got mild pain for which they went to private health facility, there doctor asked us for ultrasound. After checking ultrasound report doctor asked us to admit her in evening and told us that baby will be delivered either at night or in morning. Water break at 3:00 pm on XX and they went to the same hospital in evening and got admitted there. Second day on XX at 12 O’clock she gave birth to baby girl. Baby was normal, she was shifted at ward around 1:30 pm and she had tea and biscuit at 2:00 pm in noon. Around 2:30 she suddenly torn her clothes and asked for her husband. Her mother in law went to nurse and asked to call a doctor but nurse got angry and told her doctor has gone and will come. Again she went to doctor, doctor told nothing has happened to your daughter in law and told “I have not eaten anything from morning let me eat something”. I will give you in written that nothing will happen to your daughter in law. When doctor came after having lunch at that time she had fallen unconscious. As soon as doctor come there they had shifted her to ICU and started compressing her chest fast. She died at 5 pm in evening, after that they kept her body at YY (tertiary care) hospital and told we will not do post mortem if her parents sign the document. Her parents came and signed the document but still they have done the post mortem, but nobody at her home wants post-mortem neither her parents and nor her in laws.

**UID 53**

This was her 3rd pregnancy. Earlier she had one baby girl and had one miscarriage. Card was made but multivitamin tablets were not consumed. One injection of TT was given at civil dispensary. She went to her paternal house in ABC city during four months. She used to go to private hospital for checkups in ABC city. During 4th month she had fever and cough. She also had swelling of legs. Fever was off and on. She used to go to private doctor for medicine, but she didn’t recover. After that doctor told her that they have to admit her. 20 days before her death she got admitted in private hospital as she had high fever and severe cough. Medicine did not work. Then her in laws decided to take her to a ZZ (tertiary care) hospital for treatment. She was brought there by ambulance from ABC city on XX at 8:00 pm and admitted. She had high B.P so operation was suggested but B.P was very high, so they started giving medicine and injection to bring the B.P down. She was fine till 11 at night suddenly at 12 her B.P got low and was not coming to normal, so she was not operated. That morning at 12:30 on XX she died.

**UID 54**

This was her first delivery. She had made her card from a civil hospital. She had two injections of TT and iron folic acid tablets. She had her all the test done along with ultrasound. There was no problem. Patient went to a private hospital on XX as labor pain started. Water break at 4:00 am in morning. They had diagnosed PIH and were immediately referred to a YY (tertiary care) hospital along with the doctors in ambulance. She was admitted there, they told us about low Hb. Pain was continuous throughout the day and second day in morning on XX she gave birth to baby boy at 5:00 am. After 2 hours of delivery there was severe bleeding.  Doctor also injected glucose and blood to her. Because of severe bleeding she died. Baby also died. Baby was born through normal delivery.

**UID 55**

This was her first delivery. She had her card made from civil dispensary. Two injections of TT and Iron folic acid tablets were given. Tests were done on time and everything was normal. On XX, date was given for delivery, but when we went to WW (secondary care) hospital for checkup doctor told us to come on XX. They reached at 9:30 am on XX and she got admitted. She was admitted there for 3 days. On XX date, doctor told us that baby had passed stool inside mother’s stomach, so she has to be operated. Doctor did operation on XX at 4:00 pm and baby boy was delivered. At 6:00 pm both mother and baby were out of operation theatre till that time both were healthy, she talked whole night to her parents. Everything was fine till morning, in morning she got 3 hiccups and she died there. Doctor was called and doctors tried to save her and she could not be saved.

**UID 56**

This was her 6th baby. ANC card was prepared from civil dispensary. Two injections of TT and Iron folic acid tablets were given. Baby was delivered full term on XX at civil hospital through normal vaginal delivery. All the tests were done and were normal. On XX she asked doctor for Tubectomy. After that on XX at 3:00 she was operated for the same. At 4:00 she was out of operation theatre and everything was fine. At 4:30 she told about pain in chest, Doctor at civil hospital had checked her and on XX at 10:00 am in morning she was referred to YY (tertiary care) hospital. Doctor were also there with her in ambulance. She was admitted there and injected glucose and blood and then referred to another ZZ (tertiary care) hospital on XX at 11:30 pm. Doctor gave oxygen to her and told her relative that she has to operate again to open the stitches during tubectomy. On XX she was operated again at 4:00 am in morning. She died on same day at 9:53 am immediately after coming out of operation theatre.

**UID 57**

This was her second delivery. Previously she had a five-and-a-half-year-old boy. She was 8-month pregnant.TT-1 and two other injections were taken in civil dispensary and she also had tablets. She used to go for check up every month. She was normal. Suddenly she got fever and cough and she went to a private clinic for checkup. She took medicine from there, after one day her fever was okay but breathing problem got worse. She was taken to a WW (secondary care) hospital on XX at 1:30 pm for checkup, she was admitted there for one and a half hour and was referred to YY (tertiary care) hospital due to unavailability of ventilator. We took her to YY (tertiary care) hospital. We reached at 3:30, there doctor treated her and around 7:30 in evening they referred her to ZZ (tertiary care) hospital, as they also did not have ventilators. She was taken to ZZ (tertiary care) hospital and we reached at 8:00 pm and she was taken to labor room, there no one was allowed to go inside, and doctor gave sample to her husband for tests and told him that they do not have ventilator. Patient died at 11:00 pm at night. Doctor told us that her heart beat is low, and she need ventilator for survival, but we do not have ventilator.

**UID 58**

This was her 3rd delivery. She had made her card from local dispensary. Two injections of TT and Iron folic acid tablets were give used to go for regular checkups. All the test and ultrasound were also done. Everything was fine. On XX date labor pain started after that on XX date, in morning at 10:00am they went to civil hospital there they consulted a doctor who told him that it’s time for the delivery. Doctor took her to labor room and told us that operation has to be done as baby was not normal. They did not inform us that major operation has to be done and took signature of her husband. At 1:00 doctor started the operation and at 4:00 she was taken to labor room from operation theatre. Baby boy was born. She saw her husband and baby after coming out. After one hour at 5:00 she got backache. Her husband called doctor and informed her. Doctor told pain killer was injected in glucose and she will be fine after some time. Doctors were trying to control the pain. When pain was not under control they referred her to a ZZ (tertiary care) hospital. Their doctor told us that she is dead.

**UID 59**

This was her 8th delivery. First two babies died, they were also baby boy. Now she has 5 alive children. Her LMP period was XX. She didn’t have iron folic acid tablets. Only one injection of TT booster was received on XX. She had only one antenatal visit on XX. Neither she had any problem during her pregnancy nor B.P, diabetes or any other disease. Her pregnancy was full term i.e. 39 weeks, her Hb was 9 gm that was tested in dispensary. Labor pain started around 8:30 pm in evening, her husband came back at 8:30 pm then she told him about her labor pain and asked him to take her to hospital. Then around 12am in night her husband took her civil hospital. Their doctor gave her Glucose drip and referred her to YY (tertiary care) hospital around 12:30 they reached. She was immediately admitted there, and baby was delivered through normal vaginal delivery. Around 2:30 am at night her husband saw her sitting on floor and helped her to sit on bed. then everybody was trying to hold her but she was pushing ever body, at that time glucose drip and blood pipe were attached to her. After delivery doctor did not told her husband that she delivered still birth. After long time doctor told her husband about baby. Then her husband told doctor that baby is already gone and requested to save his wife. She was bleeding severely, her husband got faint after seeing her bleeding for this reason her husband went outside for 10 minutes. He came back after 10 minutes and try to hold his wife’s legs as she was pushing everyone. Her husband held her and doctor gave her injection so that she can be calm and don’t push anyone. she got calm after injection. Ambu bag was applied on her mouth and her husband was called to press the ambu bag, after some time her husband was sent to bring some medicine etc. around 6:20 in the morning doctor told him that his wife is no more. she was registered in civil dispensary and she was advised to have proper checkup and about consumption of iron folic acid tablets by health workers of dispensary but she every time told that i don’t want to deliver my baby here. I will go to my village for delivery.

**UID 60**

This was her 2nd pregnancy. Before this she has a live son, who born normal. She got all her ANC checkups done from civil dispensary. She took both TT injections and iron tablets were also consumed. When she was about 5-6 months pregnant she got fever for which her husband immediately took her to a YY (tertiary care) hospital.  After examination, doctors admitted her. Kept her under observation for some days. Her husband said fever was coming and going. Doctors keep her giving paracetamol. One day before death she had difficulty in breathing, so doctors gave her oxygen. Then since next morning she was uncomfortable because she was having difficulty in breathing.  In the evening she started breathing hard then her husband told the doctor. And doctor replied rudely that straighten the bed and start oxygen.  Husband did the same. Even then she was not feeling well. Then husband again went to tell it to the doctor and doctor was not there, but nurse was there. When told the nurse, she said give oxygen.  Angrily husband went back and at night he saw that she is falling pale and cold. Then he called doctor and started shouting. When doctors arrived, she was dead. According to husband doctors didn’t bother at all. Only junior doctors used to come specialists never came.

**UID 61**

This was her 2nd pregnancy. After 1 month she went to ABC city. There she lived for 4-5 months. She didn’t take any card or injection.  One ultrasound was done in the 8th month. On XX date at 9.15pm at night slight labor pain started. At 9.45 they took her to civil hospital. They checked and said baby’s heart beat is missing. Hospital people referred us to tertiary hospital. Baby got delivered and nurse told that baby is fine and bring clothes for baby. But after 15 minutes she told that baby expired and told family that she is serious, and bleeding is not stopping and referred to another ZZ tertiary hospital.  We reached there around 12 am-1am. hospital people told operation will be done. Operation was performed at night and they said anything can happen during operation. She was kept on ventilation for whole day. In afternoon she started talking. She got vomiting and loose motion. Then in the evening they took the mother for operation and said operation will take 2-3 hours. But it took only 1 hour. As they came out of operation theatre the mother died after 15 minutes. Hospital people said bleeding was not stopping. Her sister told that mother’s bleeding continued till death.

**UID 62**

This was her 1st pregnancy. One and a half year had passed since she got married. All tests were done from a ZZ tertiary care hospital. Iron tablets were also consumed, and 2 TT injections were also taken. In the 8th month they went for ultrasound to private and they said there is some problem with baby. They sent to the ZZ tertiary hospital. When ultrasound was done in there and they said baby is not passing urine because of which pressure is going on baby’s kidney. They advised to go for abortion. She and her husband agreed. Then hospital people conducted tests for 15-20 days and then called for abortion.  After some days, one morning, Sonia fell down of vertigo. Immediately they went to civil hospital, where they gave injections and said take her to ZZ tertiary care hospital. But she said she is fine now. So, they came home. Next day she again suffered from vertigo then they immediately came to hospital, then she said she is not fine. She admitted there for 3 days they gave her medications and on 4th day conducted delivery. Baby was born dead. Delivery was normal. After delivery She didn’t regain consciousness. Oxygen was given for 3 days after delivery. Then on the day when Sonia died she got fever at XX date at night. Sister in law gave her cold packs. After 1 hour she died.

**UID 63**

Three years has passed since her wedding. Her 3 children were wasted one remained for 3 months and then wasted and rest 2 were wasted at two months’ time.  This was the 4th time and this time she died. Her in laws used to give her medication as they didn't want child from her at that time. Her mother touched their feet so that they send her with her mother and her mother would take care of her during this pregnancy. Her mother sought treatment for her for the past 1 year. She was not able to conceive, so she got private treatment from a ABC city. Then she had conceived, and mother made her receive all the treatment keeping ANC in mind. all treatment was conducted by both private and government hospitals. TT injections were given, ultrasounds were done, and she also consumed the red (iron folic acid) tablets. During 9 months of pregnancy she didn’t have any problem, not even headache. Her card was prepared from civil hospital they used to call her on every Tuesday and Thursday for checkup. Nisha’s 10th month has started so she went to civil hospital for checkup and they said she has high BP and prescribed a test from YY (tertiary care) hospital.  Then we took her to YY (tertiary care) hospital on the same day, on XX date they admitted her. At YY (tertiary care) hospital they gave medication for pain to her. After some time, she delivered a girl normally. But baby didn’t cry, and they kept her in glass. After 7 days baby died. 3 hours after delivery she also died. Doctors didn’t tell us what happened to her. In fact after delivery doctors told us to bring clothes for baby and her and give her something to eat and give her tea. When we brought tea, doctors scolded us that you are thinking about tea. When we saw both her legs were tied with the cloth and injections were given repeatedly to her in the waist. After 3 hours she had died. Later one doctor told that she took tension and her tubes bursted.

**UID 64**

She got married on XX date. she conceived two months after her marriage. She had problem of cough previously. She had undergone treatment several times and she has been to private doctor also, but her cough did not get normal. She was suffering from dry cough. She got pregnant on XX and we took her to dispensary for checkup and card was also made there. Two injections of TT and iron folic acid tablets and calcium tablet were also given to her in dispensary. She took all the prescribed tablets. Two ultrasounds were also done in 3rd and 5th months respectively. she didn’t tell us about anything. We also send her to WW (secondary care) hospital for checkup. She used to tell doctors in WW (secondary care) hospital that she is fine. On XX pain started but she was alone at home then she called someone from neighbor. Neighbored came along with dai but before they came she had already delivered a baby girl. Dai cut her umbilical cord. At 8:15 baby girl was delivered and then we also came back to our home. We went for our work first then we took her to WW (secondary care) hospital for giving her injection. Both the girl and mother were given injection. She came to her brother house on XX on occasion of Bhaiduj but she got cough and difficulty in breathing, and we took her to hospital again on XX. Their doctor immediately referred her to another ZZ tertiary hospital. Doctors checked her and operated her on neck on XX, but she did not recover. They were checking her response by applying pen on her feet, but she didn’t respond. Other day on XX she died. Baby was 1.7 gm in weight; she is absolutely normal, rest we don’t know much because she was at her father’s home and they told us that she had cough and difficulty in breathing. She used to get breathing problem whenever she had severe cough. Doctor told us that she had high BP.

**UID 65**

She was married for 1 year. This is her second marriage. She had her first marriage in other state. She had 3 daughters from her first marriage 13, 11 and 8 years old and she had a still birth after 3 daughters five years ago. This was her 5th pregnancy. Her first husband had already died. She met her second husband one and half years ago. Her present husband didn’t know much about her. She was working as sweeper at the same hospital and when she had a still birth. doctor had strictly told her not to have any more baby as there is risk of her life. But she had not told her second husband about this. She was HIV positive. She had conceived after 2 months of her marriage. We got to know about pregnancy in second month and we had made her card from civil dispensary near to her home dispensary, they had also called me, but she had not told me anything. She even didn’t tell me about whether she had any injection or not. She had 3 ultrasound checkups during 5th, 8th and 9th month. When she had her entire test done during 5th month then her husband also went along with her to ZZ tertiary care hospital, then he got to know that she was HIV positive. Her husband told that after marriage she used to eat anything and vomit immediately. When he used to ask her about her problems she did not tell anything to him. She also got medicine from there, she used to take them. During 6th month of pregnancy he got to know that her liver was dead. She was also taking medicine for this. During 6th month he noticed redness in her eyes whenever she woke up. They also went to another hospital during 5th month of her pregnancy, when they saw their card and they suggested them to continue her treatment in ZZ tertiary care hospital. She used to tell him that she went for checkups regularly. During 9th month of her pregnancy she had swelling of her hand, feet and face. During 9th month when she had blood test done it was reported that she had only 4 gm of Hb. On XX date, in the morning she complained of having pain. They reached labor room around 9:00 am by autorickshaw and she delivered a baby girl through normal delivery at 9:45 am. She was given 8 bottles of glucose and 5 packets were yellow. Severe bleeding was also there after delivery due to this she had low Hb. Doctor told him that they had to stay there for few days until her Hb increases. But she kept on insisting that she wanted to go home as her children were alone at home. On XX date, they came back at home by their own will (left against medical advice).  Doctor had taken written consent from them that they were going by their own against the will of doctors. She had no secretion of milk after delivery, baby was bottle fed. Baby weight was 2 kg. On XX at 5 am she had vomiting. Her husband took medicine from local chemist.  She got normal after taking medicine, she had food after some time and she slept. Around 9-10am her husband left for his duty and he came back at 5:30 pm in evening and he called her, but she didn’t reply. When he tried to wake her up, she was already dead. He called his neighbors to check her and she was dead. Her daughter was playing outside the house they did not saw her mother. The baby girl that she delivered also died after 2 months.

**UID 66**

She was married from past 3 years. She had 2-year-old baby boy who is all right and delivered through normal delivery. This was her second pregnancy. She knew about her pregnancy from beginning. She had made her card from a WW (secondary care) hospital. She had two injection of TT and all the prescribed iron folic acid tablets. Two ultrasounds were done in the 3rd and 7th month respectively, both the ultrasound reports were normal. During pregnancy she had cough since 7th month.  She had her checkup done from WW hospital and doctor told us that it will subside after delivery. She used to take medicine but did not recover. Two and a half year ago she had nodule in her right armpit.  There was secretion of watery pus from the armpit when it was pressed. It was all right before pregnancy but during pregnancy secretion of pus was there. We had her checkup in WW hospital but they have-not done any test. She had pain in the nodule during pregnancy. One day during her 8th month of pregnancy she had pain on XX date at 7:00 pm then we took her to hospital by auto between 7:30 to 8:00 pm. She was admitted there and next day on XX date at 3:40 am she had given birth to baby girl through normal delivery. We told doctor about her pain so after 3rd day of delivery, X ray was done in same hospital. And reports of X-ray suggest that nodule had invaginated towards her breast. She was all right after delivery for 4-5 days. Only she had severe weakness I used to carry her from one place to another. One day on XX date around 2:00 pm she had difficulty in breathing and only oxygen was given in WW hospital, and was referred to ZZ (tertiary care) hospital around 2:35 pm and we took her immediately to that hospital through ambulance. There was no doctor with us. Her Hb was 5gm at that time. Then she was put at ventilator in ZZ hospital gynae ward and medicine was given. She had more problems with that medicine. They have taken blood for test despite of having low Hb. They were taking blood full of syringe continuously at the interval of 1 hour because of this only her condition got severe. She was given 3 bottles of glucose, and then on last stage doctor asked us to bring blood, around 9:00am on XX date when we brought blood she was dead. Death time was 10-11 am on XX date. Cause of death according to the ZZ hospital doctors: she had problem of tuberculosis, doctors had only told us this, but WW hospital people did not tell us about that she had TB, if we had knowledge about the TB we would have surely gone for its treatment. They have told us that might be she was having TB problem, but they were not sure. It was looking like that ZZ hospital people were doing research rather than treatment. Her baby also died in the XX month at the age of 2 months.

**ID67**

Five years passed since she got married, she also got pregnant 2 years back. But had a miscarriage in XX month because of severe bleeding. At the time of this baby it was known from the beginning that she was pregnant. She got her card prepared from civil dispensary in 3rd month. 2 TT injections were also taken. One was given on XX date and other on XX date. According to her husband she also consumed red (iron folic acid tablets) tablets. When the ultrasound was done doctors said baby was inverted.  We did many ultrasounds. One was done 2 days before delivery. Ultrasound reports used to come normal, but doctors said our baby is inverted. Discharge of water was throughout her pregnancy. Color of discharge was white. She got checkup at civil hospital and they gave tablets to eat and insert but of no use. One day on XX at 10pm in night slight pain occurred then at 1.30 pm on XX we took her to a YY tertiary hospital by auto. They said this is not our patient take her to either WW (secondary care) hospital or other civil hospital (first referral unit).  YY hospital people checked mother and they have slight idea that baby is not in normal position. Our baby also not moved much in stomach (move 2-3 times a day). Then we reached WW hospital approximately at 2.30 in night by auto. Their doctor said blood is deficient and it will be transfused. But they didn't transfused blood. Pain continued but they didn't give glucose. Then doctor called when pain started on XX at 9am. and said maternal uterine tissue has increased in size. That has to be cut, operation has to be done. And said baby's heart beat has also decreased. Our baby was absolutely fine, what doctor said that baby’s heartbeats are less there was nothing like that. They gave baby to us and mother was sent to the bed. All of us were having fun, she also. On XX at 6 pm in the evening she asked for water. Then as the time passed she started behaving like kids. She started behaving crazy.  She slapped doctors and also said bad words to us. At 8 pm she had an urge to defecate and we helped her. Then doctor transfused 2 glucose and said let us know if there is any problem. On XX at 2 am in night she had vomiting then we told the doctor and they gave her a sedative injection. But she didn’t fell asleep. Then doctor didn’t give her any treatment. Then when the next shift doctor came they were busy having fun.  After calling many times they came and at 4.30 said she is serious. Nothing will happen here take her another ZZ (tertiary care) hospital. They took half an hour to make the decision and didn’t give any treatment to the patient in the meanwhile. They just ask for disprin but didn’t give it to her. ECG was also done but everything was normal, but her BP was high. And they said she has jaundice. On XX at 10.15 pm we took her to PGI by ambulance. There they gave her a good checkup and said she had high BP because of which vessels of her brain ruptured because of which she started behaving abnormally. And because of lack of oxygen her kidney swelled up. And her kidney failed, and her heart stopped beating. Then they said there is no life in her, but we will try again. Then they made her breath through machines, but nothing happened and they gave their reply. She died on XX at 2.20pm. According to doctors cause of death-cardio respiratory arrest.

**UID 68**

She was married 5 years ago. She had 3-year-old baby boy. This was her second pregnancy. She got to know about her pregnancy in second month. She had done pregnancy test at home. After that she had her card made in third month of her pregnancy in civil dispensary. She had two injection of TT in 3rd and 5th months respectively and 100 iron folic acid tablets. All other tests were done in hospital. All reports were all right. Ultrasound was done in 5th month and was normal she had no problem during her pregnancy except she had a low B.P; it all started in 5th month. She used to go to WW (secondary care) hospital for her checkups. She used to go to WW hospital and local civil dispensary whenever doctor called her. On XX she had problem in breathing and was uncomfortable. She was taken to hospital on XX at 4 pm by auto. She had her checkup done there but they were not able to diagnose and referred her to another ZZ (tertiary care) hospital. We took ambulance from hospital and reached a ZZ hospital emergency at 8:30. After reaching there we were asked why we brought her to emergency and were instructed to take her in OPD. We told them we don’t have time and we were referred from WW hospital, so they had admitted her. But after the tests were done she was referred to ATR (Assessment Treatment and Rehabilitation) as she was having Swine flu.  She had her treatment in ATR. Neither they told us much about her condition nor did they allow us to meet her frequently. They even don’t inform us about the baby. She delivered still birth on XX. baby was a female. Doctor called us and told that she had a still birth. May be due to overdose of medicine baby died. She was having treatment for swine flu, but doctors did not inform us anything. Then on XX at 4:00 she died. After that doctor told us that she had kidney failure and having difficulty in breathing. I have seen XX 2-3 times after delivery she was lying unconscious. Maybe she had severe bleeding.
